# Unrestrained poly-ADP-ribosylation provides insights into chromatin regulation and human disease

**DOI:** 10.1016/j.molcel.2021.04.028

**Published:** 2021-05-20

**Authors:** Evgeniia Prokhorova, Thomas Agnew, Anne R. Wondisford, Michael Tellier, Nicole Kaminski, Danique Beijer, James Holder, Josephine Groslambert, Marcin J. Suskiewicz, Kang Zhu, Julia M. Reber, Sarah C. Krassnig, Luca Palazzo, Shona Murphy, Michael L. Nielsen, Aswin Mangerich, Dragana Ahel, Jonathan Baets, Roderick J. O’Sullivan, Ivan Ahel

**Affiliations:** 1 Sir William Dunn School of Pathology, University of Oxford, Oxford OX1 3RE, UK; 2 Department of Pharmacology and Chemical Biology, UPMC Hillman Cancer Center, University of Pittsburgh, Pittsburgh, PA, USA; 3 Translational Neurosciences, Faculty of Medicine and Health Sciences, University of Antwerp, Antwerp, Belgium; 4 Laboratory of Neuromuscular Pathology, Institute Born-Bunge, University of Antwerp, Antwerp, Belgium; 5 Molecular Toxicology Group, Department of Biology, University of Konstanz, 78457 Konstanz, Germany; 6 Neuromuscular Reference Centre, Department of Neurology, Antwerp University Hospital, Antwerp, Belgium; 7 Proteomics Program, Novo Nordisk Foundation Center for Protein Research, Faculty of Health and Medical Sciences, University of Copenhagen, Blegdamsvej 3B, 2200 Copenhagen, Denmark; 8 Present address: Institute for the Experimental Endocrinology and Oncology, National Research Council of Italy, 80145 Naples, Italy; 9 These authors contributed equally; 10 Lead contact

## Abstract

ARH3/ADPRHL2 and PARG are the primary enzymes reversing ADP-ribosylation in vertebrates, yet their functions *in vivo* remain unclear. ARH3 is the only hydrolase able to remove serine-linked mono(ADP-ribose) (MAR) but is much less efficient than PARG against poly(ADP-ribose) (PAR) chains *in vitro*. Here, by using ARH3-deficient cells, we demonstrate that endogenous MARylation persists on chromatin throughout the cell cycle, including mitosis, and is surprisingly well tolerated. Conversely, persistent PARylation is highly toxic and has distinct physiological effects, in particular on active transcription histone marks such as H3K9ac and H3K27ac. Furthermore, we reveal a synthetic lethal interaction between ARH3 and PARG and identify loss of ARH3 as a mechanism of PARP inhibitor resistance, both of which can be exploited in cancer therapy. Finally, we extend our findings to neurodegeneration, suggesting that patients with inherited ARH3 deficiency suffer from stress-induced pathogenic increase in PARylation that can be mitigated by PARP inhibition.

## In brief

Prokhorova et al. show that accumulation of PARylation, but not MARylation, is highly toxic to the cell, perturbing DNA synthesis, chromatin organization, and transcription and eventually leading to PARP-dependent cell death. This underlies the synthetic lethality between PARG and ARH3 in cancers and the development of neurodegeneration in ARH3/ADPRHL2-deficient patients.

## Graphical Abstract

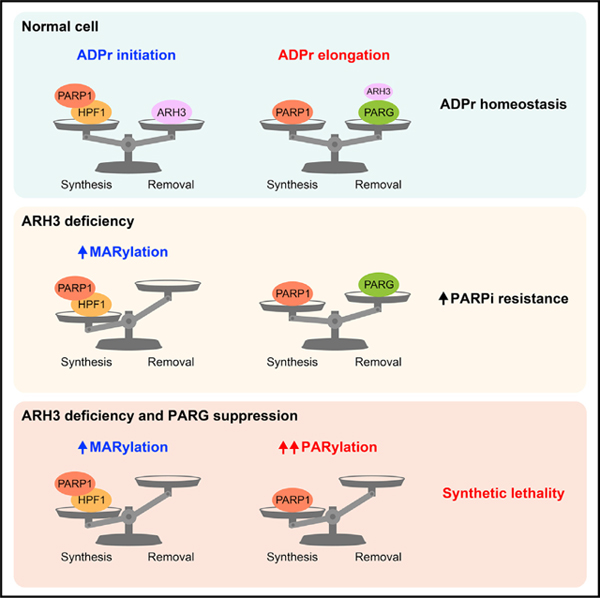

## INTRODUCTION

ADP-ribosylation (ADPr) is a reversible post-translational protein modification (PTM) that regulates numerous processes, including DNA damage repair and chromatin remodeling (Gupte et al., 2017; Liu et al., 2017; Palazzo et al., 2017). The ADP-ribose moiety is transferred from NAD^+^ onto an acceptor amino acid on the target protein, with the simultaneous release of nicotinamide. Poly(ADP-ribose) polymerases (PARPs) constitute the major family of ADP-ribosylating enzymes, which consists of 17 members in human cells (Azarm and Smith, 2020). PARP1, the founding and most studied enzyme in the family, accounts for 85% of cellular ADPr upon DNA damage (Amé et al., 1999; Chen et al., 2018). PARP1 rapidly binds to DNA ends at the sites of damage and modifies itself, histones, and other proteins with mono- and poly(ADP-ribose) (MAR and PAR, respectively). These modifications, which promote and control DNA repair, occur predominantly on serine residues (Bonfiglio et al., 2017; Buch-Larsen et al., 2020; Hendriks et al., 2019, 2021; Leidecker et al., 2016; Palazzo et al., 2018) and, as such, require an accessory factor HPF1 for efficient synthesis (Bilokapic et al., 2020; Bonfiglio et al., 2017; Gibbs-Seymour et al., 2016; Hendriks et al., 2021; Suskiewicz et al., 2020).

The timely removal of DNA damage-induced ADPr is required to prevent trapping of proteins recruited in a PAR-dependent manner and to allow access for the downstream DNA repair factors, as well as nucleotide recycling (Liu et al., 2017). PAR glycohydrolase (PARG) and ADP-ribosyl hydrolase 3 (ARH3/ADPRHL2) are the two human enzymes capable of cleaving the O-glycosidic bond between ADPr subunits within PAR chains *in vitro* (Lin et al., 1997; Oka et al., 2006). PARG is the most active PAR hydrolase (Fontana et al., 2017) and is essential for mouse and *Drosophila* development (Hanai et al., 2004; Koh et al., 2004), with PARG knockout (KO) resulting in only rarely surviving flies exhibiting elevated PARylation and progressive neurodegeneration (Hanai et al., 2004). PARG is unable to remove protein-linked MAR (Fontana et al., 2017; Slade et al., 2011), and its activity is reduced with decreasing chain length down to low levels for oligomers of two to four ADPr units (Barkauskaite et al., 2013). ARH3 activity against PAR chains is one to two orders of magnitude lower than PARG (Fontana et al., 2017; Oka et al., 2006). Nevertheless, ARH3 contributes to the reversal of DNA damage-induced PARylation in certain contexts (Mashimo et al., 2013) and is responsible for the removal of serine-linked MAR synthesized by the PARP1/HPF1 complex under both basal and DNA damage conditions (Fontana et al., 2017; Palazzo et al., 2018). Although *ARH3*-deficient mice are viable and do not exhibit phenotypic defects without external challenges, *ARH3*-deficient mice and mouse embryonic fibroblasts (MEFs) show increased sensitivity to high levels of hydrogen peroxide (H_2_O_2_), which can be rescued by PARP inhibition (Mashimo et al., 2013, 2019). Furthermore, loss-of-function mutations in *ARH3* were recently linked to the pathogenesis of several neurodegenerative phenotypes in human patients (Danhauser et al., 2018; Ghosh et al., 2018; Mashimo et al., 2019). This implies that, similar to PARG, ARH3 protects cells from the accumulation of ADPr, although the underlying mechanisms are still unknown.

The emerging role of ARH3 in the control of cellular MARylation and, at least to some extent, PARylation levels suggests that both ARH3 and PARG complement each other in maintaining ADPr homeostasis. However, despite a great interest in PARP1 as a target for cancer therapy and the arising therapeutic potential of PARG inhibition (Chen and Yu, 2019; Lord and Ashworth, 2017; Nagashima et al., 2020; Pillay et al., 2019; Slade, 2020), the relative contribution of ARH3 and PARG in regulating the levels of ADPr in human cells remains unclear.

Here, we show that MARylation is constantly generated on chromatin throughout all stages of the cell cycle, including mitosis, but its enrichment in ARH3-deficient cells is surprisingly well tolerated. Then, we address the separation of function between ARH3 and PARG and describe the synergistic effect of combined ARH3 loss and PARG suppression on the levels of PARylation. We distinguish between the two distinctly regulated steps in ADPr reaction, namely, initiation, which is catalyzed predominantly by PARP1/HPF1 and reversed by ARH3, and elongation, which is performed mainly by PARP1 alone and counteracted by PARG. We further demonstrate that excessive PARylation is highly toxic to the cell, disrupting chromatin modification and transcription states, perturbing DNA synthesis, telomere maintenance, and cell proliferation, and eventually leading to PARP-dependent cell death. Consequently, we identify a synthetic lethal interaction between ARH3 and PARG and also reveal the loss of ARH3 as a new mechanism of PARP inhibitor (PARPi) resistance. Altogether, our data highlight the potential for ARH3 as a novel drug target and a biomarker for cancer cell sensitivity to PARP and PARG inhibition. Finally, we extend the significance of the timely hydrolysis of ADPr beyond cancer, proposing a role for ARH3 in the prevention of the detrimental accumulation of PARylation during the onset of neurodegeneration.

## RESULTS

### Loss of ARH3 results in the accumulation of non-toxic chromatin MARylation throughout the cell cycle

**Figure 1.**
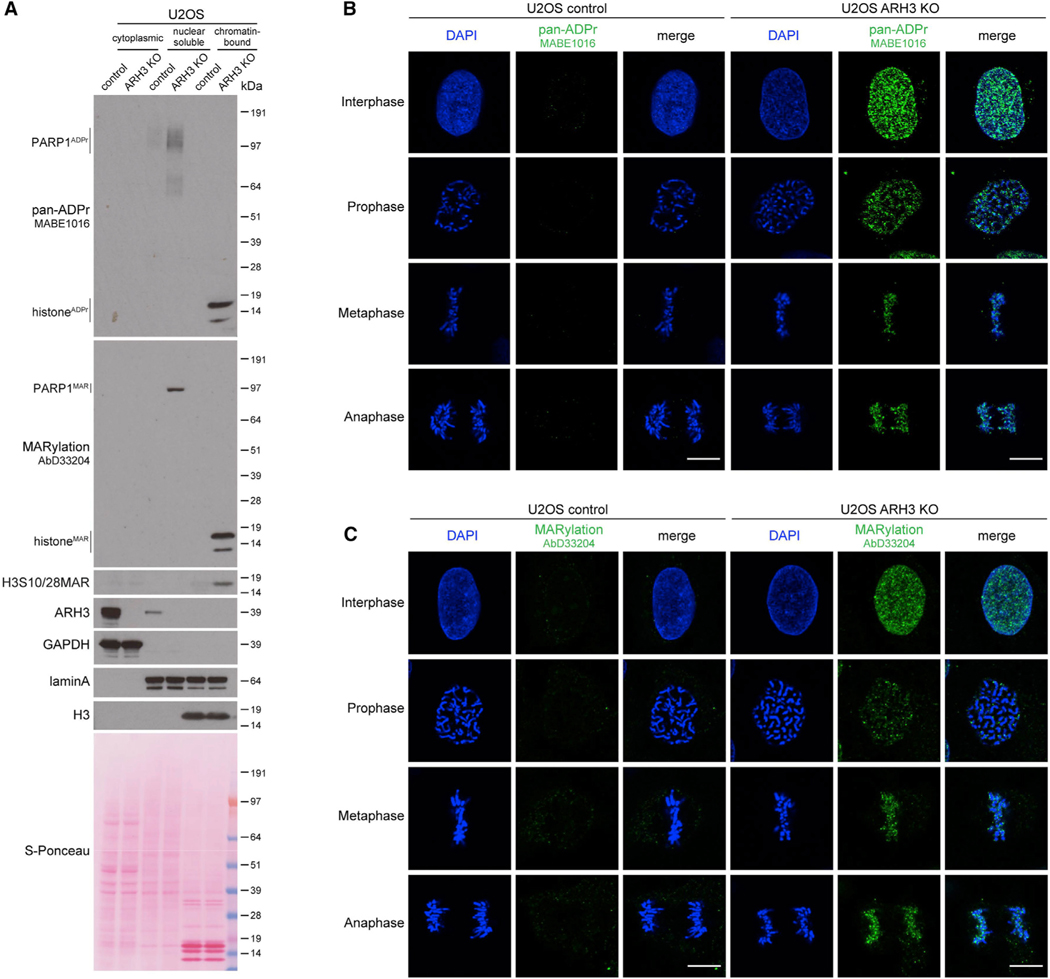
Loss of ARH3 leads to the enrichment of chromatin-associated MARylation throughout the cell cycle (A) Cells were subjected to subcellular fractionation. ADPr signals were analyzed using western blotting. (B and C) Pan-ADPr (B) and MARylation (C) signals were detected by immunofluorescence in detergent pre-extracted cells at the indicated cell cycle stages. Scale bars, 10 μm. See also Figure S1.

The loss of ARH3 hydrolase results in the accumulation of serine-ADPr not only in response to DNA damage but also in untreated conditions (Fontana et al., 2017; Hanzlikova et al., 2020; Palazzo et al., 2018). To better characterize the housekeeping role of ARH3 in the reversal of endogenous ADPr, we assessed the levels and localization of ADPr in control and ARH3-KO U2OS cells at different stages of the cell cycle. Subcellular fractionation followed by western blotting using anti-pan-ADPr reagent, which binds to all forms of cellular ADPr (Gibson et al., 2017), showed strong accumulation of ADPr in ARH3-KO but not in control cells, and the modification was predominantly detected on chromatin (Figure 1A). A similar increase in chromatin ADPr was observed using anti-MARylation AbD33204 and anti-histone H3 S10/28-linked MAR antibodies (Bonfiglio et al., 2020; Figure 1A). Immunofluorescence analysis in cells pre-extracted with detergent prior to fixation to retain chromatin-bound proteins but remove cytoplasmic and nuclear soluble components also showed the enrichment of ADPr in ARH3-KO cells and, surprisingly, revealed that ADPr persisted not only in interphase but even on condensed chromatin throughout different stages of mitosis (Figures 1B and 1C). Subcellular fractionation following cell cycle synchronization confirmed these observations (Figures S1A and S1B). The signals were lost upon small interfering RNA (siRNA)-mediated *HPF1* knockdown (Figures S1C–S1E), which together with a recent mass spectrometry study (Hendriks et al., 2021) confirms that ADPr in ARH3-KO U2OS cells is specifically enriched at serine residues.

Surprisingly, the loss of ARH3 resulting in the marked enrichment of MAR is well tolerated as ARH3-KO cells proliferate similarly to control cells (Figure S1F) and normally progress through multiple cell cycles. This is unexpected given that MARylation is enriched on chromatin, even during mitosis, and that persistent ADPr may affect canonical histone modifications critical for cell proliferation (Bartlett et al., 2018).

### Both ARH3 and PARG protect the cell from excessive PARylation, with ARH3 removing initial serine-ADPr attachments and PARG hydrolyzing their elongation products

**Figure 2.**
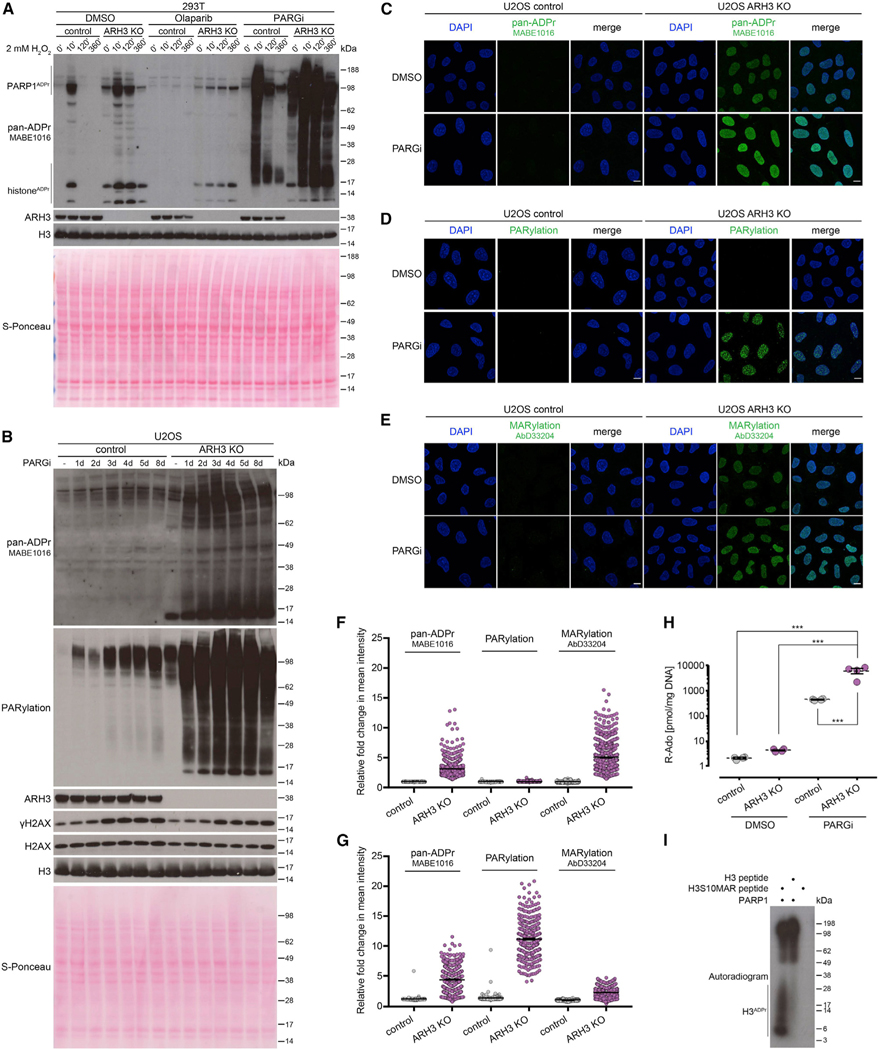
Suppression of PARG activity leads to the accumulation and persistence of PARylation in ARH3-deficient cells (A) Cells were pre-treated with DMSO, 10 μM olaparib, or 10 μM PARGi for 1 h followed by 2 mM H_2_O_2_ treatment for the indicated time in the presence of the drugs. ADPr signals were analyzed using western blotting. (B) Cells were treated with DMSO for 8 days or with 25 μM PARGi for the indicated number of days. ADPr and γH2AX levels were analyzed using western blotting. (C–E) Levels of pan-ADPr (C), PARylation (D), or MARylation (E) were analyzed using immunofluorescence in detergent pre-extracted cells treated with DMSO or 25 μM PARGi for 6 days. Scale bars, 10 μm. (F and G) Quantification of (C)–(E) for 6 day DMSO (F) or 25 μM PARGi treatment (G). Data represent fold change in mean intensity per nucleus relative to DMSO-treated control cells and are shown as mean ± SEM; at least 300 cells were analyzed per condition. (H) Levels of PAR in U2OS cells treated with DMSO or 25 μM PARGi for 4 days were quantified by UPLC-MS/MS analysis. Ribosyl-adenosine (R-Ado) is representative for the overall content of PAR. Data are shown as mean ± SEM, n = 4; ***p < 0.001 (one-way ANOVA followed by Tukey post-test). (I) Radioactive ADP-ribosylation assay of unmodified H3 peptide or H3 peptide with S10-linked MAR (H3S10MAR). See also Figure S2.

We then decided to explore ARH3 function under exogenous stress conditions, using a short treatment with H_2_O_2_ that is known to cause oxidative stress and subsequent DNA damage. Western blotting with anti-pan-ADPr reagent was used to assess the levels of ADPr. Consistent with our previous findings (Fontana et al., 2017; Hanzlikova et al., 2020; Palazzo et al., 2018), ARH3 loss resulted in increased ADPr levels in both untreated and H_2_O_2_-treated 293T cells (Figure 2A), with the strongest signals corresponding to histone and PARP1 ADPr. H_2_O_2_-induced ADPr returned to baseline levels 2 h after treatment in control cells but remained elevated in ARH3-KO cells. A short 1 h pre-treatment with the PARP1/2 inhibitor olaparib completely blocked the H_2_O_2_-induced ADPr signal in control cells, showing that this signal is PARP1/2 dependent. Conversely, the elevated basal levels of histone and PARP1 ADPr in ARH3-KO cells persisted despite 1 h pre-treatment with olaparib (Figure 2A) but nevertheless disappear after prolonged treatment with PARPi (Hanzlikova et al., 2020).

Although PARG has higher hydrolytic activity against long PAR chains *in vitro*, ARH3 is also able to remove PAR, albeit with much lower efficiency (Fontana et al., 2017; Oka et al., 2006). To further examine whether these two enzymes cooperate in regulating cellular PARylation levels, we treated control and ARH3-KO cells with the PARG inhibitor (PARGi) PDD00017273 (James et al., 2016). PARGi treatment resulted in a dramatic increase in H_2_O_2_-induced ADPr in both control and ARH3-KO 293T (Figure 2A) and U2OS cells (Figure S2A) as detected by anti-pan-ADPr reagent and anti-PARylation antibody, indicating that PARG accounts for the hydrolysis of the majority of PARylation in response to DNA damage. However, H_2_O_2_-induced ADPr was strongly diminished in PARGi-treated control but not ARH3-KO cells at the 2 and 6 h time points (Figure 2A), suggesting that, although slower and less efficient than PARG, ARH3 also contributes to the turnover of DNA damage-induced PARylation. Of note, simultaneous suppression of ARH3 and PARG led to a substantially greater persistence of ADPr compared with their individual effects combined, suggesting that the roles of ARH3 and PARG are not redundant but rather synergistic.

Importantly, in the absence of exogenous stress, ARH3 loss resulted only in a very small enrichment of PARylation compared with control cells (Figure S2A), suggesting that indeed, endogenous ADPr in ARH3-deficient cells consists predominantly of MARylation. However, just 1 h pre-treatment of ARH3-KO cells with PARGi caused a substantial increase in PARylation levels (Figures 2A and S2A). Longer treatment resulted in a further increase in ADPr, in particular PARylation, as detected by western blotting (Figures 2B, S2B, and S2C), immunofluorescence following detergent pre-extraction prior to fixation to retain only chromatin-associated proteins (Figures 2C–2E, 2G, and S2E), and ultraperformance liquid chromatography-tandem mass spectrometry (UPLC-MS/MS) (Figure 2H). Conversely, only a small increase in PARylation was observed in control cells, even following prolonged PARGi treatment, demonstrating that not only PARG but also ARH3 plays a major role in the maintenance of endogenous PARylation levels. A similar increase in ADPr signals specifically in ARH3-KO cells was recapitulated upon siRNA-mediated *PARG* knockdown (Figure S2F), ruling out possible side effects of PARGi. Similar results were obtained following PARG inhibition and ARH3 deletion in HeLa background (Figure S2G), confirming the universality of our observations.

The above data clearly show that ARH3 contributes to the reversal of both stress-induced and endogenous PARylation. However, enrichment of MARylation but not PARylation in untreated ARH3-deficient cells (Figures 2D–2F) and the exceptional hydrolytic activity of ARH3 against serine-linked MAR *in vitro* (Figure S2H) indicate that ARH3 is critical for the reversal of initial MAR rather than of the products of its elongation. Conversely, PARG cannot remove initial serine-ADPr attachments (Figure S2H) but is the main enzyme that reverses PARylation, which is, however, composed mainly of PARP1 autoPARylation in the absence of exogenous stress when ARH3 activity is present (Figures 2B, S2F, and S2G). ARH3 also contributes to the removal of at least basal PARP1 autoPARylation and possibly shorter PAR that is detected by anti-pan-ADPr reagent but not anti-PARylation antibody (Figures 2A, 2B, S2A, S2F, and S2G). Importantly, the remarkable increase in endogenous PARylation upon simultaneous ARH3 and PARG suppression implies that ARH3 is crucial for preventing accumulation of basal PARylation, even more so than of stress-induced PARylation (Figure 2B compared with Figure S2B). The effect of the dual suppression of ARH3 and PARG on endogenous ADPr levels is markedly greater that the sum of their individual effects, again highlighting the synergy rather than simple redundancy or additivity between the two hydrolases.

Indeed, both ARH3 and PARG are required for the maintenance of low levels of cellular PARylation (Figures 2B, S2F, and S2G), and combined ARH3 and PARG activity is necessary for the complete removal of serine-linked PAR *in vitro* (Figure S2H). However, according to our data, ARH3 affects PARylation largely indirectly, by removing initial serine-ADPr attachments that can act as seeds for subsequent elongation to PAR. In the presence of PARG, these seeds persist as serine-linked MAR, and only upon PARG suppression can they become stably extended to long PAR chains. Conversely, if PARG is active but the elongation step is blocked by PARP1/2 inhibition, these initial attachments persist specifically in ARH3-KO but not control cells both under undamaged and DNA damage conditions (Figure 2A). Therefore, our data provide compelling evidence for both synergy and distinct separation of function between ARH3 and PARG.

Moreover, our results imply the existence of two separate stages in ADPr reaction, namely, initiation and elongation, with the reverse steps being controlled predominantly by ARH3 and PARG, respectively. Given that initiation of serine-ADPr by PARP1 requires a cofactor protein HPF1 (Suskiewicz et al., 2020) and that elongation of ADPr, particularly on PARP1 itself, can happen on its own and is remarkably faster than the initiation (Mendoza-Alvarez and Alvarez-Gonzalez, 1993, 1999; Naegeli et al., 1989), we suggest that although the initiation stage of ADPr reaction is performed by PARP1/HPF1, PARP1 alone is sufficient to catalyze the elongation step. In agreement with this, although PARP1 alone cannot modify histone H3 peptide *in vitro*, it can efficiently PARylate the same peptide if it was previously “primed” with an initial serine-linked MAR (Figure 2I). This further supports the existence of initiation and elongation stages in ADPr reaction with distinct regulation of both its forward and reverse steps.

### ARH3 deficiency sensitizes cancer cells to PARG inhibition and renders cancer cells resistant to PARP inhibition

**Figure 3.**
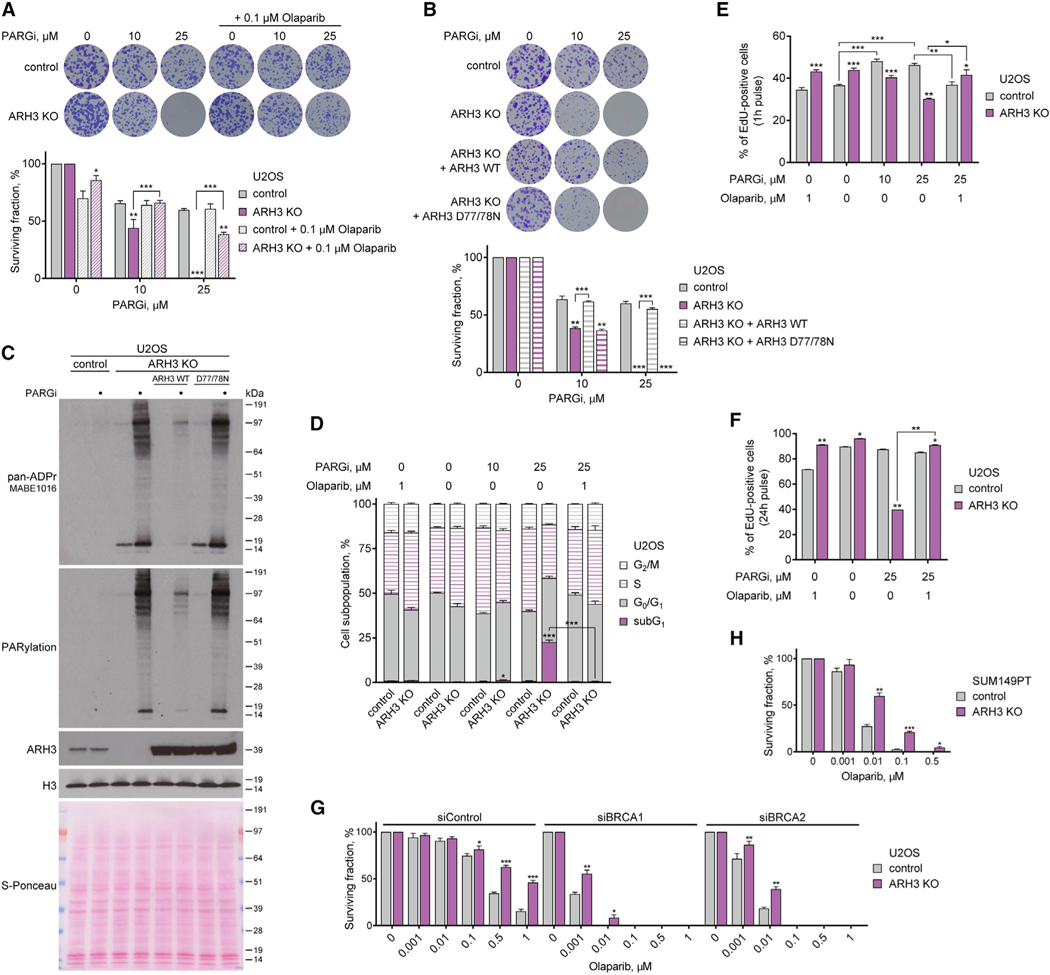
ARH3 deficiency is synthetically lethal with PARG suppression and renders cancer cells resistant to PARP inhibition (A and B) Representative images (top) and quantification (bottom) of colony formation assay with control and ARH3-KO cells (A and B) and ARH3-KO cells complemented with ARH3 WT or catalytically inactive D77/78N mutant (B) treated with DMSO or as indicated. (C) Cells were treated with 25 μM PARGi for 4 days. ADPr signals were analyzed using western blotting. (D) Quantification of cell cycle analysis by flow cytometry of EdU- and DAPI-stained cells after 6 day exposure to DMSO or indicated treatment and 1 h EdU pulse. (E and F) Cell proliferation and DNA synthesis after exposure to DMSO or indicated treatment for 6 days and 1 h (E) or 24 h (F) EdU pulse. (G and H) Quantification of colony formation assay with U2OS cells transfected with BRCA1 or BRCA2 siRNA (G) or with SUM149PT cells (H) treated with DMSO or olaparib. Data are shown as mean ± SD, n = 3 (A, B, G, and H), or as mean ± SEM, n = 4 (E), n = 2 (F); *p < 0.05, **p < 0.01, and ***p < 0.001 (two-tailed Student’s t test). See also Figure S3.

Previously, suppression of PARG activity was found to lead to the accumulation of γH2AX, a marker of DNA double-strand breaks (Chen and Yu, 2019; Pillay et al., 2019; Ray Chaudhuri et al., 2015). Consistent with these data, PARG suppression resulted in elevated γH2AX levels in both control and ARH3-KO U2OS and HeLa cells. Surprisingly, ARH3 deficiency by itself did not increase γH2AX levels, and no additional γH2AX induction was seen following simultaneous suppression of PARG and ARH3 (Figures 2B, S2D, S2F, and S2G). Despite there being no additive effect of PARG suppression and ARH3 loss on DNA break formation, we hypothesized that persistent accumulation of chromatin ADPr would be toxic to the cell. Indeed, we observed that the loss of ARH3 results in a marked sensitization of U2OS (Figures 3A and S3A) and HeLa cells (Figure S3B) to PARG inhibition by performing a long-term colony formation assay. To determine whether this is due to the accumulation of PARP-dependent ADPr, we attempted to rescue the phenotype by PARP inhibition. Addition of low concentrations of the PARP1/2 inhibitors olaparib and veliparib suppressed the toxicity of PARGi treatment in both ARH3-KO U2OS (Figures 3A and S3C) and HeLa cells (Figure S3D). Next, to confirm that the sensitivity of ARH3-KO cells to PARGi is due specifically to the loss of ARH3 enzymatic activity, we genetically complemented ARH3-KO U2OS cells with either wild-type (WT) ARH3 or D77/78N ARH3, a catalytic mutant that completely lacks the ability to remove ADPr (Fontana et al., 2017; Oka et al., 2006). Complementation with WT but not D77/78N ARH3 rescued PARGi sensitivity (Figure 3B) and restored the reversal of PARGi- and H_2_O_2_-induced ADPr (Figures 3C and S3E). Altogether, these results reveal a synthetic lethal interaction between ARH3 and PARG caused by extreme accumulation of endogenous PARylation due to the absence of their hydrolase activities. Furthermore, these data demonstrate that although chromatin-associated MARylation is well tolerated throughout multiple cell cycles, accumulation of PARylation is highly toxic.

We then sought to determine whether persistently elevated PARylation in PARGi-treated ARH3-deficient cells would disrupt cell cycle progression. Interestingly, PARGi treatment or ARH3 loss alone caused an increase in the proportion of S-phase cells in both U2OS (Figures 3D, 3E, and S3G) and HeLa cells (Figures S3I and S3H) as measured by EdU incorporation and DAPI staining using flow cytometry. PARGi treatment in ARH3-KO cells substantially reduced the number of cells undergoing S phase but markedly induced the accumulation of subG1 population (Figures 3D and S3H), consistent with the observed synthetic lethality between PARG and ARH3. Importantly, these defects were completely rescued following the addition of olaparib (Figures 3D and 3E), highlighting their dependency upon PARP1/2 activity. Additionally, a longer 24 h EdU pulse-labeling assay showed a further reduction in DNA synthesis and cell proliferation in PARGi-treated ARH3-KO U2OS cells that was again rescued by olaparib (Figure 3F). Altogether, whereas loss of either PARG or ARH3 activity alone results in enrichment of S-phase cells, exposure of ARH3-KO cells to PARGi markedly decreases DNA synthesis and cell proliferation. A quantitively greater and qualitatively different outcome of the combined suppression of ARH3 and PARG compared with their individual inactivation again confirms that their roles are synergistic. This is consistent with the model in which ARH3 and PARG each mainly perform one of the two steps needed for the prevention of excessive PARylation, namely, the reversal of initial serine-ADPr attachments and the hydrolysis of their subsequent elongation into PAR, respectively.

Notably, we also observed a significant decrease in cell proliferation in olaparib-treated control compared with ARH3-KO U2OS cells (Figures 3E and 3F). Indeed, a modest but statistically significant reduction in control cell survival was already observed with low concentration of olaparib using a long-term colony formation assay in both U2OS (Figure 3A) and HeLa cells (Figure S3D). These data suggest that the loss of ARH3 activity might confer resistance to PARPi toxicity similarly to PARG downregulation, which was recently reported to be one of the mechanisms of PARPi resistance in cancers due to a resultant increase in PARylation (DeWeirdt et al., 2020; Gogola et al., 2018). Therefore, we additionally explored the effect of ARH3 loss on PARPi sensitivity in the context of *BRCA1/2* deficiency in which PARPis show particularly promising results in clinics. Although, as expected, siRNA-mediated knockdown of *BRCA1* or *BRCA2* resulted in a dramatic sensitization of U2OS cells to olaparib, cells with ARH3 deficiency showed a substantial resistance to the treatment compared with control cells (Figures 3G and S3J). Moreover, the loss of ARH3 increased PARPi resistance in triple-negative breast cancer (TNBC) SUM149PT (*BRCA1* mutant, *BRCA2*-WT) and SUM159PT (*BRCA1/2*-WT) cells (Figures 3H, S3K, and S3L). Similar to U2OS cells, siRNA-mediated knockdown of *BRCA1* or *BRCA2* sensitized SUM159PT cells to PARP inhibition. However, ARH3-KO cells were significantly more resistant to the treatment than control cells (Figures S3M and S3N). Overall, these data confirm that in addition to sensitizing cells to PARG inhibition, loss of ARH3 confers PARPi resistance in different types of cells, including *BRCA1/2*-deficient cancers.

### PARylation interferes with telomere elongation by ALT mechanism and globally affects chromatin modification and transcription profiles

**Figure 4.**
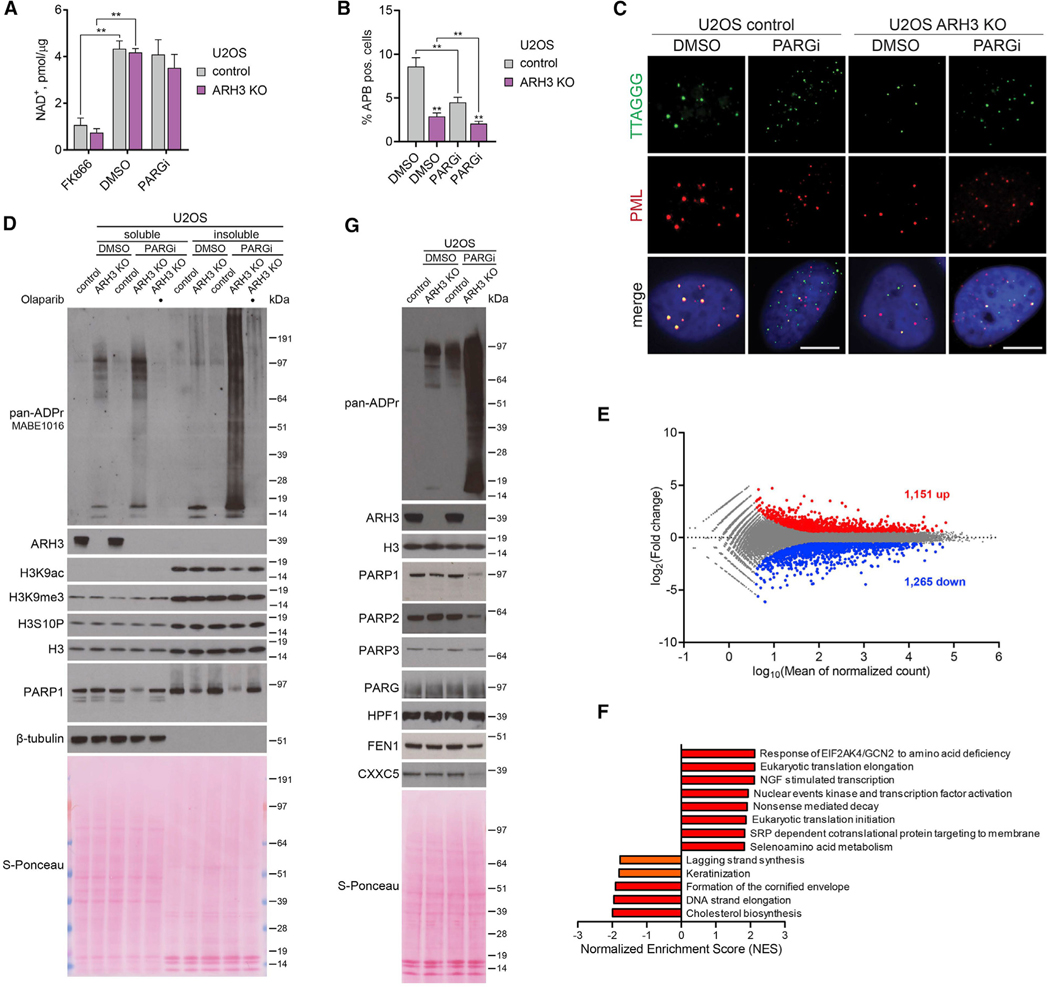
Simultaneous loss of ARH3 and PARG activity causes dysregulation of chromatin modification and transcription profiles (A) NAD^+^ quantification assay in cells treated with DMSO or 25 μM PARGi for 4 days or 10 nM FK866 for 1 day. Data are shown as mean ± SD, n = 2; **p < 0.01 (two-tailed Student’s t test). (B and C) Quantification (B) and representative images (C) of ALT-associated PML bodies (APBs) after DMSO or 5 μM PARGi treatment for 72 h. Data in (C) are shown as mean ± SEM, n = 5; at least 1,300 cells were analyzed per condition; **p < 0.01 (two-tailed Student’s t test). Scale bars, 5 μm. (D and G) Cells were treated with DMSO or 25 μM PARGi for 6 (D) or 4 days (G). ADPr and protein levels were analyzed using western blotting. (E)MA plot showing differentially expressed genes (upregulated in red, downregulated in blue) in ARH3-KO U2OS cells treated with 25 μM PARGi for 4 days against DMSO control. n = 3, adjusted p < 0.05, absolute fold change > 1.5. (F) Summary of significantly upregulated and downregulated pathways from gene set enrichment analysis (GSEA) in ARH3-KO U2OS cells treated with 25 μM PARGi for 4 days against DMSO control. n = 3, q < 0.05 (in red), q < 0.1 (in orange). See also Figure S4.

Next, we further addressed the mechanisms underlying the cytotoxicity of excessive ADPr. PARP1 is one of the major cellular consumers of NAD^+^, and PARP1 activation was shown to reduce cellular NAD^+^ level under DNA damage conditions (Cohen, 2020). We thus examined if PARGi-induced accumulation of ADPr in ARH3-KO U2OS cells is accompanied by NAD^+^ depletion. Although there was a slight trend toward a reduction in NAD^+^ level, the difference was not statistically significant (Figure 4A). In contrast, treatment with FK866, a highly specific inhibitor of nicotinamide phosphoribosyltransferase (NAMPT), an essential enzyme in the biosynthesis of NAD^+^, dramatically reduced NAD^+^ levels in both control and ARH3-KO cells, confirming the sensitivity of the NAD^+^ assay used. These results argue against NAD^+^ depletion as the major cause of cell death upon PARG inhibition in ARH3-KO cells.

Furthermore, PARG activity was recently demonstrated to play a critical role in telomere extension through the alternative lengthening of telomeres (ALT) mechanism in cancer cells, including U2OS (Hoang et al., 2020). Therefore, we decided to explore whether the additional loss of ARH3 would further impair this mechanism that is crucial for the survival of these cancer cells. A defining characteristic of ALT mechanism is the association of a subset of telomeres within specialized PML bodies forming ALT-associated PML bodies (APBs) (Yeager et al., 1999). Quantification of APBs by immunofluorescence combined with fluorescence *in situ* hybridization (FISH) is routinely used to assess the extension of telomeres in ALT cancer cells (Hoang and O’Sullivan, 2020). As before, we found that PARG inhibition substantially decreased APB levels in U2OS cells. Interestingly, ARH3 loss alone resulted in a similar effect, and combined PARG inhibition and ARH3 deficiency further decreased APB formation (Figures 4B and 4C). This indicates that both ARH3 and PARG are involved in telomere maintenance in ALT cancer cells and highlights dysregulation of telomere maintenance as an additional mechanism underlying the synthetic lethality of ARH3 and PARG suppression in ALT cancer cells.

Next, given the dramatic accumulation of histone ADPr upon PARGi treatment in ARH3-deficient cells, we examined whether neighboring histone modifications, in particular acetylation, may be consequently affected. Histone H3S10 is one of the main sites of cellular serine-ADPr, which was shown to be mutually exclusive with acetylation of H3K9 (H3K9ac) (Bartlett et al., 2018; Larsen et al., 2018; Liszczak et al., 2018). Indeed, prolonged exposure to PARGi resulted in a marked decrease in H3K9ac levels in ARH3-KO but not control U2OS (Figures 4D, S4A, and S4B) and 293T cells (Figure S4D). Importantly, the observed reduction in H3K9ac was rescued by olaparib, confirming the mutually exclusive relationship between acetylation and PARylation. On the other hand, the levels of H3K9me3, H3S10P, and total H3 remained stable across all conditions (Figures 4D and S4D). Additionally, transient 24 h overexpression of H3-GFP in control and ARH3-KO 293T cells allowed us to monitor H3 modification in newly incorporated nucleosomes, showing that in this sensitized background, ARH3 deficiency alone already results in a substantial decrease in H3-GFP K9ac levels (Figures S4C and S4D), as was recently reported (Hanzlikova et al., 2020). Importantly, this effect was also observed for endogenous histone H3 co-immunoprecipitated with the nucleosomes that incorporated H3-GFP. Exposure to PARGi caused an even greater reduction in K9ac of H3-GFP and associated endogenous H3. Additionally, we observed a decrease in endogenous H3K27ac, but not in H3K27me3 levels (Figure S4D).

Taking into account that H3K9/27ac marks are associated with active transcription regions (Lawrence et al., 2016) and a recent report that ARH3-deficient patient fibroblasts acquire alterations in gene expression (Hanzlikova et al., 2020), our results imply that unrestrained PARylation caused by simultaneous ARH3 and PARG suppression could affect cellular transcription. Of note, the decrease in H3K9/27ac levels in 293T cells occurred even prior to an increase in γH2AX formation (Figure S4D), further suggesting that dysregulation of chromatin modification rather than DNA damage induction contributes to the toxicity of excessive PARylation in cells lacking both ARH3 and PARG activity. Moreover, we detected a decrease in protein levels of PARP1 and PARP2 (Figures 4D, 4G, and S4D) that by itself could contribute to transcriptional dysregulation (Kim et al., 2020; Páhi et al., 2020). Indeed, by performing RNA sequencing and differential gene expression analysis, we observed significant changes in the transcriptome of ARH3-KO cells following PARG inhibition with 748 upregulated and 827 downregulated protein-coding and 403 upregulated and 438 downregulated non-protein-coding genes (Figures 4E and S4G). In comparison, only 147 protein-coding genes were found to be differentially expressed in ARH3-deficient patient fibroblasts (Hanzlikova et al., 2020). Although we did not detect significant changes in *PARP1* and *PARP2* mRNA expression, indicating that their protein levels are reduced post-transcriptionally, we were able to confirm a decrease in FEN1 and CXXC5 expression both on mRNA and protein levels (Figure 4G). To identify the pathways that may contribute to the toxicity of excessive PARylation in PARGi-treated ARH3-KO cells, we performed gene set enrichment analysis (GSEA) (Mootha et al., 2003; Subramanian et al., 2005). As shown in Figure 4F, we found that 13 cellular pathways were significantly affected by PARG inhibition in ARH3-KO cells, including downregulated lagging strand synthesis and DNA strand elongation pathways, in which FEN1 is one of the central players.

To elaborate on the role of the post-transcriptional reduction in PARP1 levels in PARGi-treated ARH3-KO cells, we assessed the effect of PARP1 overexpression on cell survival. In agreement with our findings that PARP inhibition rescues PARGi sensitivity of ARH3-KO cells by preventing ADPr, overexpression of PARP1 further exacerbated PARGi-induced toxicity and ADPr accumulation (Figures S4E and S4F). Of note, although we detected considerable decreases in protein levels of PARP1/2 enzymes that can catalyze PARylation, we did not observe changes in protein levels of PARP3 (Figure 4F), which can only perform MARylation (Azarm and Smith, 2020). Collectively, we assume that the observed post-transcriptional reduction in PARP1/2 protein levels in PARGi-treated ARH3-KO cells is the adaptive consequence rather than the cause of the phenotype and could serve as a survival mechanism of escaping the toxicity of excessive PARylation and developing resistance to PARG and/or ARH3 inhibition.

In conclusion, in certain cellular models the enrichment of MARylation resulting from ARH3 deficiency could already lead to some alterations in histone acetylation and gene expression, which in themselves are not toxic to the cell. However, excessive accumulation of PARylation upon simultaneous loss of ARH3 and PARG activity leads to a substantially greater dysregulation of histone acetylation and transcription profiles, which likely contributes to its toxicity. The extent of these defects thus depends upon the length and abundance of ADPr and can be specific to both the location and type of modification. Collectively, we identified a number of different pathways affected by unrestrained PARylation in PARGi-treated ARH3-deficient cells. The effects of the combined suppression of ARH3 and PARG were qualitatively different and many fold greater than of their individual inactivation not only in the case of accumulation of PARylation and resulting cell death but also dysregulation of DNA synthesis, histone modification, and gene expression profiles, as well as post-transcriptional downregulation of PARP1/2. This confirms the synergy rather than simple redundancy or additivity between the two hydrolases and highlights the importance of the proposed two-step model of ADPr reaction in the maintenance of cellular homeostasis.

### Loss of ARH3 function in primary patient fibroblasts results in PARylation accumulation and PARP-dependent cell death

**Figure 5.**
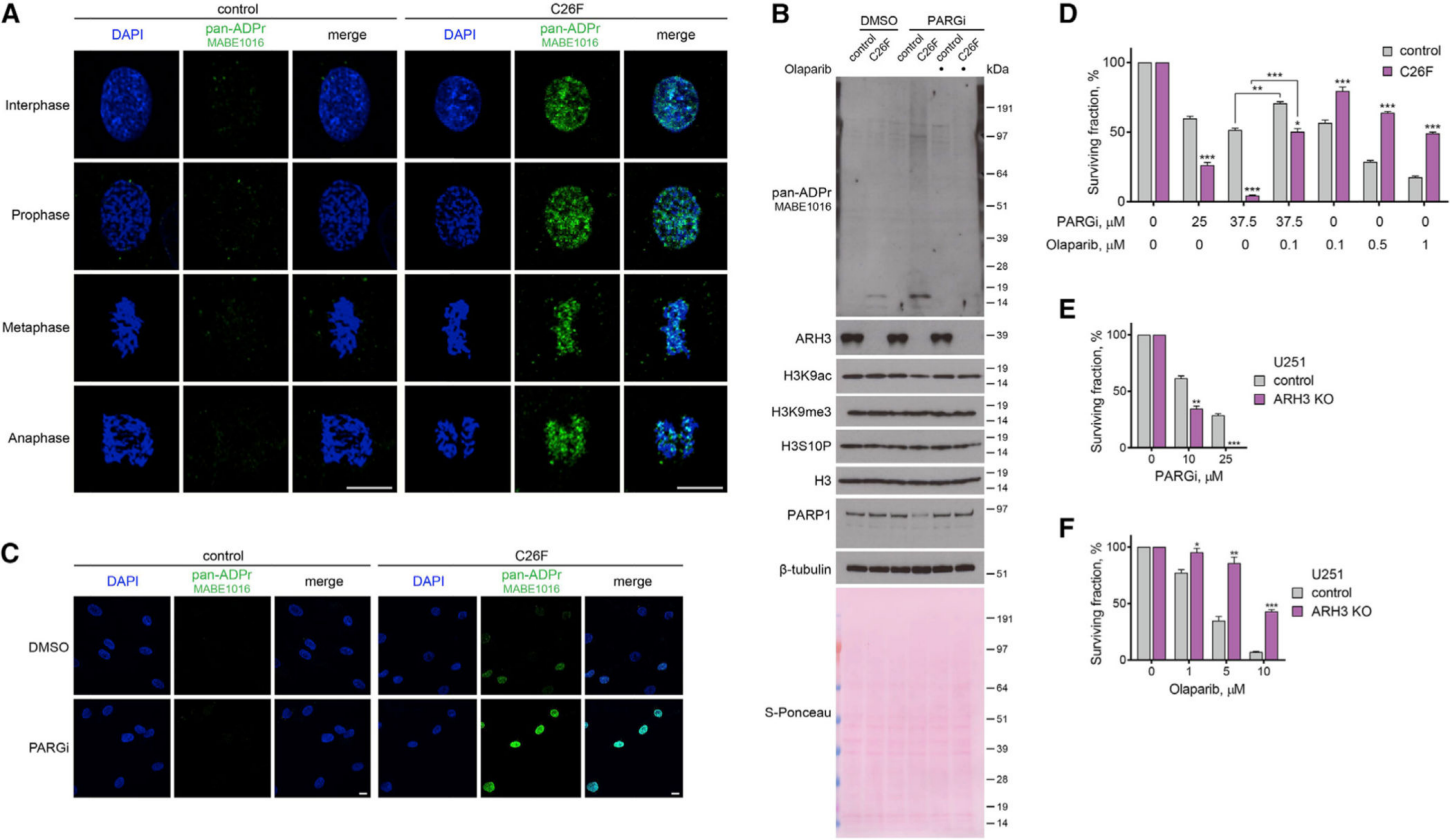
Loss of ARH3 activity in patient-derived primary fibroblasts and glioblastoma cells leads to the accumulation of ADPr, increased PARGi sensitivity, and PARPi resistance (A) Pan-ADPr signals were detected using immunofluorescence in detergent pre-extracted control and ARH3 C26F mutant patient cells at different cell cycle stages. Scale bars, 10 μm. (B) Cells were treated with DMSO, 25 μM PARGi or 25 μM PARGi, and 1 μM olaparib for 10 days. ADPr, H3 modification, and PARP1 levels were analyzed using western blotting. (C) Levels of pan-ADPr were analyzed using immunofluorescence in detergent pre-extracted cells treated with DMSO or 25 μM PARGi for 4 days. Scale bars, 10 μM. (D) Quantification of crystal violet assay with cells treated with DMSO or as indicated. (E and F) Quantification of colony formation assay with U251 cells treated with DMSO or as indicated. Data are shown as mean ± SD, n = 3; *p < 0.05, **p < 0.01, and ***p < 0.001 (two-tailed Student’s t test). See also Figure S5.

In recent years, several patients were reported to harbor pathogenic *ARH3* mutations causing progressive neurodegeneration (Danhauser et al., 2018; Ghosh et al., 2018; Mashimo et al., 2019). We obtained primary fibroblasts from a patient homozygous for a newly identified pathogenic missense mutation in ARH3 NM_017825: c.77G > T (p.Cys26Phe) (C26F) and from an unaffected healthy control individual. The C26F mutation caused a substantial reduction in ARH3 protein levels indicative of a loss-of-function allele (Figure S5A), possibly due to an increase in Van der Waals volume upon C26F mutation being incompatible with the correct folding of the protein. Similar to ARH3-KO cells, we detected the enrichment of endogenous chromatin ADPr at all stages of the cell cycle (Figures 5A), as well as increased basal and PARGi-induced total ADPr, in C26F mutant compared with control primary patient fibroblasts (Figures S5A, 5B, and 5C). Moreover, the C26F cells were sensitive to PARG inhibition (Figure 5D), and we detected a reduction in PARP1 and H3K9ac but not H3K9me3 and H3S10P levels following PARGi treatment (Figure 5B). Importantly, all of the above phenotypes were rescued by olaparib. Additionally, we genetically complemented the C26F cells with either WT ARH3 or catalytically inactive D77/78N ARH3 and observed the rescue of the phenotypes only with WT ARH3 (Figure S5B). Notably, similar to ARH3 loss, C26F mutation rendered cells more resistant to olaparib treatment (Figure 5D), suggesting that PARPi administration could be a safe therapeutic measure to prevent stress-induced accumulation of toxic PARylation in ARH3-deficient patients. To further support the potential relevance of our findings to the cell types more related to neurodegeneration, we assessed the effect of ARH3 loss on the sensitivity of glioblastoma U251 cells to PARG and PARP inhibition. Again, we observed increased ADPr and reduced PARP1 levels in ARH3-KO compared with control cells following PARG inhibition (Figure S5C). Moreover, loss of ARH3 rendered U251 cells more sensitive to PARGi and resistant to olaparib (Figures 5E and 5F).

Overall, these findings confirm that the loss of ARH3 function results in the enrichment of endogenous chromatin ADPr, in itself not toxic but “priming” the cells for subsequent accumulation of detrimental PARylation, eventually leading to PARP-dependent cell death. Importantly, our data show that ARH3 deficiency not only renders cells more sensitive to PARGi but also confers resistance to PARP inhibition. This could potentially be exploited in the treatment of patients with *ARH3* loss-of-function mutations by allowing the safe use of higher doses of PARPis in ameliorating the progression of neurodegeneration.

## DISCUSSION

Our understanding of the functions of PARPs in DNA repair and the mechanistic details underpinning the action of PARPis have been significantly advanced by recent progress in the field (Azarm and Smith, 2020; Lord and Ashworth, 2017). However, the role of ADPr hydrolases and the consequences of their suppression remain largely uncharacterized despite their clear importance for ADPr homeostasis. Here, we describe the functional synergy and identify a synthetic lethal interaction between the major ADPr hydrolases ARH3 and PARG.

**Figure 6.**
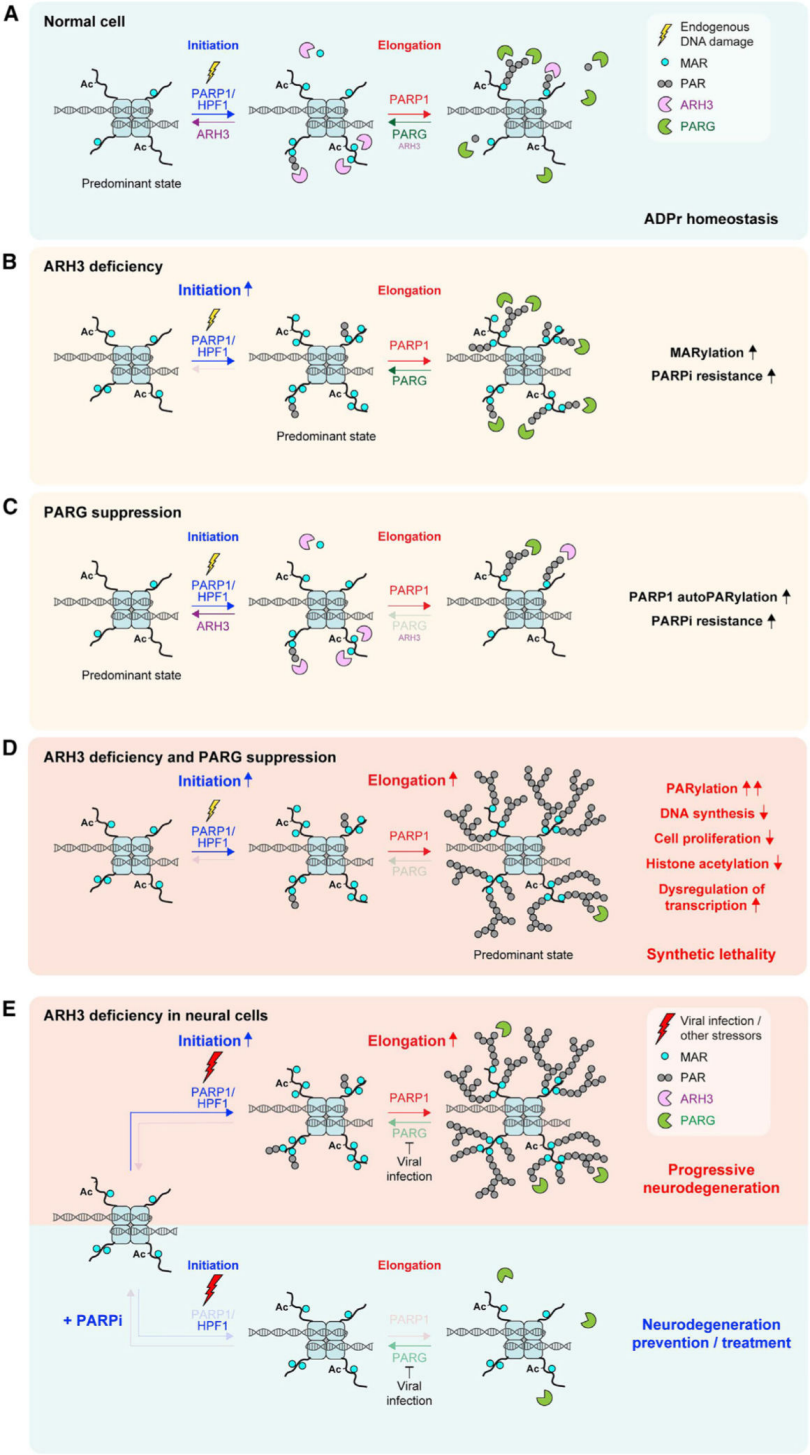
Models of two-step ADPr reaction and stress-induced pathogenesis in ARH3-deficient neurodegeneration patients (A) ADPr reaction consists of distinctly regulated initiation and elongation steps. Initiation of ADPr at serine residues requires PARP1 activation (e.g., by endogenous DNA damage) and a cofactor protein HPF1. Elongation of initial ADPr attachments can be performed by PARP1 alone. ARH3 and PARG hydrolases predominantly reverse initiation and elongation steps, respectively. (B) ARH3 deficiency results in the accumulation of MAR and short PAR initiation sites, which cannot be efficiently elongated, because of the presence of PARG activity. (C) Because of the presence of ARH3 that removes the initiation sites necessary for the subsequent elongation step, suppression of PARG results only in a slight enrichment of elongation products, composed mainly of PARP1 autoPARylation but not chromatin-associated PARylation. (D) Combined ARH3 deficiency and PARG suppression results in both initiation and elongation steps proceeding without reversal. The accumulated initiation sites are uncontrollably extended to long PAR chains, which are toxic to the cell and eventually lead to PARP-dependent cell death. (E) ARH3-deficient neural cells accumulate MAR and short PAR initiation sites, and are “primed” for rapid elongation upon encountering additional stressors, in particular viral infection, that could specifically downregulate PARG, promoting pathogenic accumulation of PARylation and thus neurodegeneration (top). PARPi treatment can alleviate stress-induced neurodegeneration in ARH3-deficient neural cells by preventing the formation of both initiation and elongation products (bottom).

In unperturbed conditions, cellular ADPr is very transient and its levels are very low (Figure 6A), but this endogenous ADPr can be detected if its reversal is suppressed. Consequently, using ARH3-deficient cells, we were able, for the first time, to demonstrate that serine-linked MAR and possibly short PAR is generated at all stages of the cell cycle and is largely associated with chromatin (Figure 6B). Indeed, many chromatin and chromatin-binding proteins were identified as serine-ADPr substrates by mass spectrometry (Bonfiglio et al., 2017; Buch-Larsen et al., 2020; Hendriks et al., 2019, 2021; Larsen et al., 2018). Thus, non-toxic suppression of ARH3 can be a valuable tool to study the many regulatory functions of protein ADPr under undamaged conditions.

Combined with previous findings (Abplanalp et al., 2017; Fontana et al., 2017; Hanzlikova et al., 2020; Hendriks et al., 2021; Palazzo et al., 2018), our data confirm that ARH3 is the main hydrolase of endogenous serine-linked MAR (Figures 6A and 6B), while PARG swiftly removes long PAR chains (Figures 6A and 6C) that are composed largely of PARP1 autoPARylation in an unstressed environment but could also come from the DNA replication-associated events, namely unligated Okazaki fragments (Hanzlikova et al., 2018). Critically, whereas elevated MARylation in ARH3-deficient cells is well tolerated, combined ARH3 deficiency and PARG suppression results in the accumulation and persistence of PARylation that is highly toxic to the cell and has distinct (patho)physiological effects (Figure 6D). These effects include dysregulation of histone modification and gene expression profiles, perturbation of DNA synthesis and cell proliferation, and together eventually lead to PARP-dependent cell death. Therefore, our study discriminates between the physiological consequences of cellular MARylation and PARylation and reveals that the observed toxicity of excessive PARylation underlies the synthetic lethality between ARH3 and PARG.

Fundamentally, our findings allow us to distinguish, for the first time, between the two distinct steps in cellular ADPr reaction, namely, initiation and elongation (Figure 6A), in a way comparable with some other PTMs (i.e., ubiquitination). Although in the case of ADPr, both steps are catalyzed by PARP1, initiation of ADPr at serines residues requires PARP1 activation (e.g., by DNA damage) and a cofactor protein HPF1 (Suskiewicz et al., 2020) and is reversed by ARH3 (Fontana et al., 2017). In certain cellular contexts, other enzymes that possess MARylation activity (e.g., SIRT6) (Mao et al., 2011), could also contribute to the initiation of ADPr. Then, PARP1-mediated elongation of ADPr, particularly on PARP1 itself, can happen on its own and is remarkably faster than the initiation (Mendoza-Alvarez and Alvarez-Gonzalez, 1993, 1999; Naegeli et al., 1989), but is efficiently counteracted by PARG. Therefore, ARH3 loss alone mainly results in the enrichment of serine-linked initiation sites in the form of MAR and possibly short PAR, but long PAR chains do not accumulate, because of the presence of PARG (Figure 6B). Conversely, inhibition of PARG activity alone leads to persistence of only elongation products, which to some extent increases PARP1 automodification levels but largely does not affect global or especially chromatin ADPr, as ARH3 cleaves the initial seeds essential for PARP1-mediated elongation (Figure 6C). Combined ARH3 loss and PARG suppression unleash both initiation and elongation steps of ADPr, resulting in extreme levels and persistence of PARylation (Figure 6D). Overall, we suggest that initiation and elongation of ADPr are separate events in both their synthesis and reversal, and the combined activities of both hydrolases create two stages of defense, providing a secure mechanism of protection against accumulation of ADPr beyond a critical threshold in vertebrates.

The observed cytotoxicity of PARylation suggests that it may also provide an additional mechanistic strategy in the complex control of cellular metabolism in higher organisms. Accordingly, vertebrates have evolved numerous PAR-binding factors that were shown to play a critical role in the maintenance of genome stability, chromatin organization, gene expression, and protein degradation (Ahel et al., 2009; Chou et al., 2010; Luijsterburg et al., 2016; Mehrotra et al., 2011; Teloni and Altmeyer, 2016). However, the role of endogenous ADPr in the regulation of chromatin state is still poorly understood. Previously, we described crosstalk between histone serine-ADPr and canonical PTMs under DNA damage conditions, in particular showing that H3 ADPr is mutually exclusive with neighboring acetylation but not methylation marks (Bartlett et al., 2018). Here, we extend these findings to physiological conditions, demonstrating that chromatin MARylation and possibly short PARylation could affect histone acetylation but not methylation in certain scenarios, such as on newly incorporated nucleosomes, as was recently reported (Hanzlikova et al., 2020). The lesions associated with DNA replication, namely, unligated Okazaki fragments, were suggested as the primary source of endogenous chromatin ADPr marks (Hanzlikova et al., 2020). However, it could also be that these modifications serve a previously undescribed housekeeping function similar to canonical histone modification marks. Indeed, with 9,029 ADPr sites, 82% of which are at serine residues, identified on proteins involved in various cellular pathways (Buch-Larsen et al., 2020), it is very likely that even the effects of ARH3 loss alone would be pleiotropic. Accordingly, following simultaneous suppression of ARH3 and PARG, we observed dramatic changes in histone acetylation and numerous other cellular pathways, which were affected on both transcriptional and post-transcriptional levels.

PARG inhibition has recently emerged as a promising approach in cancer treatment, particularly in combination with DNA-damaging agents or radiotherapy (Slade, 2020). Although PARGi PDD00017273 is unsuitable for clinical studies because of poor bioavailability (Gravells et al., 2017; James et al., 2016), recently developed PARGis with improved potency and properties may prove good candidates for clinical application (Chen and Yu, 2019; Houl et al., 2019). Of note, the synthetic lethal interaction between PARG and ARH3 described above may be exploited as a novel chemotherapeutic strategy in cancers with ARH3 or PARG downregulation, in particular, including ALT cancers. Thus, developing ARH3 inhibitors would be highly beneficial for use in combination with PARG inhibition or as an alternative approach. Moreover, PARG downregulation was reported as one of the mechanisms of PARPi resistance due to the resultant increase in PARylation (Gogola et al., 2018), and PARG was identified as a top PARPi resistance gene in cancer cells (DeWeirdt et al., 2020; Gogola et al., 2018). Thus, according to our data, PARPi-resistant cancers with PARG downregulation could be targeted by ARH3 inhibitors, further highlighting the need for ARH3 inhibitor development (Drown et al., 2018; Rack et al., 2018). Of note, we also reveal the loss of ARH3 as another potential mechanism through which different types of cancer cells, including *BRCA1/2*-deficient cancers, could acquire PARPi resistance. Altogether, our results highlight the high potential of ARH3 both as a drug target and as a predictive biomarker for cancer cell sensitivity to PARP and PARG inhibition.

Finally, our findings provide novel insights into neurodegeneration in ARH3-deficient patients (Danhauser et al., 2018; Ghosh et al., 2018; Mashimo et al., 2019). Generally, defective reversal of ADPr by PARG and TARG1 hydrolases was already known to lead to neurodegenerative phenotypes (Hanai et al., 2004; Sharifi et al., 2013). Our data suggest that neural cells lacking ARH3 function have elevated levels of basal serine-linked MAR and possibly short PAR, in particular on chromatin, that persist throughout the cell cycle but are normally well tolerated. In contrast, when ARH3-deficient neural cells encounter additional stressors (e.g., viral infection or environmental stressors) (Ghosh et al., 2018; Grady et al., 2012), they may accumulate extreme levels of PARylation that shortly overwhelm cell tolerance (Figure 6E, top), disrupting chromatin modification and transcription states and eventually leading to PARP-dependent cell death. Notably, infection by herpesviruses was shown to induce PARylation by specifically downregulating PARG (Grady et al., 2012), in this way mimicking PARG inhibition. Overall, we propose that the main rationale for the pathogenesis of neurodegeneration in ARH3-deficient patients is that constitutive MARylation efficiently “primes” the cells for stress-induced PARylation rather than being toxic in itself. Moreover, in agreement with several recent reports (Danhauser et al., 2018; Ghosh et al., 2018; Mashimo et al., 2019), our data indicate that the stress-induced neural cell death in ARH3-deficient patients could be prevented by PARPi treatment (Figure 6E, bottom). Strikingly, we also show that the loss of ARH3 function renders cells resistant to PARP inhibition, suggesting that reduced PARPi toxicity would allow the safe therapeutic use of PARPis in ARH3-deficient patients with minimal side effects.

In summary, our study reveals a synergistic interaction between ARH3 and PARG, clarifying the molecular basis of cellular ADPr and distinguishing between two distinct steps in ADPr reaction, namely, initiation and elongation. We discriminate between the physiological effects of cellular MARylation and PARylation, showing that although the former is non-toxic and is constantly produced on chromatin throughout the cell cycle, the accumulation of the latter has distinct pathogenic consequences and eventually leads to PARP-dependent cell death. Moreover, we identify the synthetic lethal interaction between ARH3 and PARG, which is caused by extreme levels of PARylation, and highlight ARH3 as a promising drug target and a biomarker for cancer cell sensitivity to PARP and PARG inhibition. Furthermore, we extend the role of ARH3 beyond cancer to restraining the detrimental accumulation of PARylation in neural cells and highlight the therapeutic potential of PARP inhibition in alleviating neurodegeneration in patients with ARH3 deficiency.

### Limitations of study

Despite the clear therapeutic potential for ARH3 and PARG as drug targets and biomarkers for cancer cell sensitivity to PARP and PARG inhibition, implementation of these strategies in clinics currently faces significant challenges. To date, there are no clinically approved ARH3 or PARGis, and none of them have yet entered clinical trials. Although promising compounds targeting PARG were recently described, ARH3 inhibitors suitable for clinical trials remain to be developed. Then, to our knowledge, there are currently no ARH3 or PARG antibodies available for immunohistochemistry diagnostics, development of which is needed to allow the use of ARH3 and PARG protein levels as predictive biomarkers in oncology. Future molecular studies of proteins involved in ADPr and their cellular targets will further advance the development of current cancer and neurodegeneration therapies. However, validating their results in animal models and other model systems would be highly beneficial.

## STAR★METHODS

### RESOURCE AVAILABILITY

#### Lead contact

Further information and requests for resources and reagents should be directed to and will be fulfilled by the Lead Contact, Ivan Ahel (ivan.ahel@path.ox.ac.uk).

#### Materials availability

All research reagents generated by the authors will be made available on request from the Lead Contact.

#### Data and code availability

RNA-sequencing data generated during this study are available in the Gene Expression Omnibus repository, https://www.ncbi.nlm.nih.gov/geo (accession GEO: GSE167060). Original imaging data are available at Mendeley Data https://doi.org/10.17632/zbmchm3fz4.1.

### EXPERIMENTAL MODEL AND SUBJECT DETAILS

#### Cell culture

Human U2OS osteosarcoma (ATCC HTB-96), cervix adenocarcinoma HeLa (ATCC CCL-2), embryonic kidney 293T (ATCC CRL-3216) cells were acquired from ATCC and grown in DMEM (Sigma) supplemented with 10% FBS (GIBCO) and penicillin-streptomycin (100 U/mL, GIBCO). Human triple-negative breast cancer SUM149PT (CVCL_3422) and SUM159PT (CVCL_5423) were a gift from Ian Gibbs-Seymour (University of Oxford) and were cultured in Ham’s F-12 (GIBCO) supplemented with 5% FBS (GIBCO), HEPES (10 mM, GIBCO), Insulin (5 μg/mL, Sigma) and Hydrocortisone (5 μg/mL, Sigma). Human glioblastoma U251 cells (CVCL_0021) were a gift from Omer Dushek (University of Oxford) and were cultured in DMEM (Sigma) supplemented with 10% FBS (GIBCO) and penicillin-streptomycin (100 U/mL, GIBCO). All cell lines were cultured at 37°C with 5% CO2.

The patient-derived primary human fibroblasts were obtained from a cartilage biopsy from a patient carrying a homozygous C26F *ARH3* mutation. A control skin-derived fibroblast line was obtained from a healthy individual. Fibroblast cultures were maintained in DMEM (Sigma) supplemented with 15% FBS (GIBCO), 1% L-Glutamine (Sigma) and penicillin-streptomycin (100 U/mL, GIBCO) at 37°C with 5% CO_2_.

To induce DNA damage, cells were incubated with 2 mM H_2_O_2_ (Sigma) in DPBS with calcium and magnesium (GIBCO) for the indicated times. For PARP or PARG inhibition, cells were pretreated with 10 μM Olaparib (Cayman Chemical) or 10 μM PARGi PDD00017273 (Sigma) for 1 h, and Olaparib or PARGi PDD00017273 was added to the DPBS solution in the case of subsequent DNA damage induction.

#### Generation of cell lines

The protocol for generating ARH3 KO HeLa, SUM149PT, SUM159PT and U251 cell lines has been used previously for U2OS cells (Fontana et al., 2017). Briefly, sgRNA 210 (GCGCTGCTCGGGGACTGCGT) and sgRNA 212 (GGGCGAGACGTCTATAAGGC) were cloned into epX459(1.1), pX459 plasmid containing enhanced Cas9 (eSpCas9) v1.1. 293 T cells were transfected with control sgRNA or cotransfected with sgRNAs 210 and 212 (1:1 ratio) using TransIT-LT1 Transfection Reagent (Mirus Bio), following the manufacturer’s instructions. 24 h after transfection, the cells were selected with Puromycin (InvivoGen) for 36 h and seeded on 96-well plates at low densities (0.4 cells/well). Single colonies were propagated, and individual clones were screened by western blotting.

For the complementation of ARH3 KO U2OS cells with ARH3 WT or catalytically inactive D77/78N mutant, ARH3 cDNA was cloned from ARH3 expression construct that was a gift from Paul Hergenrother (University of Illinois) into the pDONR221 vector (Invitrogen). ARH3 catalytic mutant D77/78N was made using QuikChange Lightning Site-Directed Mutagenesis Kit (Agilent). For the complementation of ARH3 KO U2OS cells, mammalian expression ARH3 untagged pDEST12.2 (Invitrogen) constructs were generated using LR Clonase II enzyme mix (Invitrogen). Cells were plated in 6-cm dishes and transiently transfected with pDEST12.2-ARH3 WT or pDEST12.2-ARH3 D77/78N using TransIT-LT1 Transfection Reagent (Mirus Bio) according to the manufacturer’s protocol. After 24 h, the cells were transferred into 15-cm dishes and allowed to grow for 48 h in complete DMEM. After 48 h, the media was replaced with complete DMEM supplemented with 1 mg/mL G-148 solution (Sigma) for 10 days to select for resistant cells integrated with the pDEST12.2 ARH3 constructs. Once G-148 resistance single cell colonies had formed, individual clones were transferred to individual dishes. Individual cell colonies were propagated and screened for successful integration by PCR. ARH3 expression levels were analyzed by western blotting on positive colonies. For the complementation of ARH3 C26F mutant patient-derived primary human fibroblasts, mammalian expression lentiviral ARH3 untagged pLX304 (Addgene #25890) constructs were generated using LR Clonase II enzyme mix (Invitrogen). To prepare lentivirus particles, pLX304-ARH3 WT or pLX304-ARH3 D77/78N were cotransfected with packaging plasmids pCMV-VSV-G and pCMV-dR8.2 dvpr (Addgene plasmids #8485 and #8455) using Polyfect (QIAGEN) following the manufacturer’s instructions into 293T cells. Lentiviral supernatants were collected 36 h after transfection, filtered and used for infection of ARH3 C26F mutant patient-derived primary human fibroblasts in the presence of 2 μg/mL of polybrene (Sigma). After 48 h, the media was replaced with complete DMEM supplemented with 3 μg/mL Blasticidin (InvivoGen) for 7 days to select for resistant cells integrated with pLX304 ARH3 constructs. ARH3 expression levels were analyzed by western blotting.

### METHOD DETAILS

#### Cell proliferation assays

For colony formation assays, cells were plated at low densities in 6-well plates (700 cells/well for U2OS and SUM159PT cells, 800 cells/well for HeLa cells, 900 cells/well for SUM149PT and U251 cells) and grown in the indicated conditions for 11 days. Cells were fixed and stained with 0.5% crystal violet in 25% methanol for 30 min, washed with water and air-dried. For patient-derived primary human fibroblasts crystal violet assays, 1400 cells/well were plated in 6-well plates in the indicated conditions. Media containing DMSO or fresh inhibitors was replaced every 4 days and cells were processed after 18 days as described above. Quantification was performed using ImageJ/Fiji. The surviving fraction at each dose was calculated after normalization to the plating efficiency of untreated samples. Each experiment was performed in triplicates. For quantification of cell proliferation in Figure S1F, control and ARH3 KO cells were plated in 6-well plates, and were trypsinized and counted after 24 h, 48 h, 72 h, 96 h and 120 h using MOXI Z Automated Cell Counter.

#### Western blotting

The cells were lysed with Triton X-100 lysis buffer (50 mM Tris-HCl pH 8.0, 100 mM NaCl, 1% Triton X-100) supplemented with 5 μM MgCl_2_, protease and phosphatase inhibitors (Roche), Olaparib (Cayman Chemical; 1 μM for U2OS and HeLa; 2 μM for 293T cells), PARGi PDD00017273 (Sigma; 1 μM for U2OS and HeLa; 2 μM for 293T cells) and 2 μM Trichostatin A (Sigma) at 4°C. The lysates were incubated with 0.1% Benzonase (Sigma) for 30 min at 4°C, centrifuged at 14,000 rpm for 15 min, and the supernatants were collected. As we previously noted that significant amounts of histone acetylation could be lost after centrifugation, both supernatant (soluble) and pellet (insoluble) fractions were used in Figure 4D and whole cell lysates (obtained following the same protocol without the last centrifugation step) were analyzed in Figures 4G, S4C, S4D, and 5B. For subcellular fractionation in Figures 1A and S1B, subcellular protein fractionation kit (Thermo Fisher Scientific, 78840) was used according to the manufacturer’s instructions. Protein concentrations were analyzed by Bradford Protein Assay (Bio-Rad). Proteins were boiled in 1x NuPAGE LDS sample buffer (Invitrogen) with TCEP or DTT (Sigma), resolved on NuPAGE Novex 4%–12% Bis-Tris gel s (Invitrogen), and transferred onto nitrocellulose membranes (Bio-Rad) using Trans-Blot Turbo Transfer System (Bio-Rad). The membranes were blocked in PBS buffer with 0.1% Tween 20 and 5% non-fat dried milk for 1 h at room temperature, and incubated overnight with primary antibodies (1:1000, unless stated otherwise) at 4°C, followed by 1-h incubation with peroxidase-conjugated secondary anti-mouse (Agilent, P0447, 1:3000), anti-rabbit (Agilent, P0399, 1:3000) or anti-human (Bio-Rad, STAR126P, 1:3000) antibody at room temperature. Anti-PARylation rabbit antibody (4336-BPC-100) was from Trevigen. Rabbit anti-pan-ADPr (MABE1016; 1:1500), anti-histone H3 (07–690, 1:5000), and mouse anti-PARG (MABS61, 1:500), anti-PARP2 (MABE18, 1:500), anti-GAPDH (MAB374, 1:3000), anti-cyclin B1 (05–373), anti-BRCA1 (OP92, 1:500), anti-BRCA2 (OP95, 1:500) antibodies were from Millipore. Rabbit anti-ARH3/ADPRHL2 (HPA027104; 1:2000) antibody was from Atlas Antibodies. Rabbit anti-PARP1 (ab32138; 1:2000), anti-γH2AX (ab2893), anti-H3S10P (ab5176), anti-H3K9me3 (ab8898), anti-H3K27ac (ab4729), anti-β-tubulin (ab6046; 1:5000), anti-laminA (ab26300), anti-GFP (ab290, 1:3000) anti-FEN1 (ab17994), anti-cyclin A (ab32798), anti-PRC1-phospho-T481 (ab62366), and mouse anti-H3K27me3 (ab6002) and anti-Hsp70 (ab2787) antibodies were from Abcam. Rabbit anti-H3K9ac (9649), anti-H2AX (7631), anti-CXXC5 (84546), and mouse anti-cyclin E1 (4129) antibodies were from Cell Signaling. Rabbit anti-PARP3 (11289–1-AP) antibody was from Proteintech. Mouse anti-PML (sc-996) antibody was from Santa Cruz. Rabbit anti-MARylation (AbD33204, 1:500) and human anti-H3S10/28MAR (AbD33644, 1:500) antibodies were previously described in Bonfiglio et al. (2020). Custom-made rabbit HPF1 and PRC1 antibodies were previously described (Gibbs-Seymour et al., 2016; Gruneberg et al., 2006). Blots were developed using ECL (Invitrogen) and analyzed by exposing to films.

#### Cell synchronization

Control and ARH3 KO U2OS cells were plated in 10-cm dishes and subjected to double thymidine block synchronization. Cells were treated with 2.5 μM thymidine (CalBiochem) for 18 h washed with PBS and complete DMEM, and released into fresh complete DMEM for 11 h. Then, the cells were again arrested with thymidine for additional 12 h. Cells were washed as before and harvested at 4 h (S-phase), 8 h (G2-phase) and 11 h (mitosis) post-wash out time points. Asynchronous samples were grown untreated throughout the same time period.

#### siRNA transfection

siRNA transfection was performed using Lipofectamine RNAiMAX (Invitrogen) and 20 nM siRNA for the indicated time according to the manufacturer’s instructions.

#### Immunofluorescence and confocal microscopy

Cells were seeded on glass coverslips and grown in the indicated conditions. Cells were washed with PBS, when indicated pre-extracted with 0.2% Triton X-100/PBS supplemented with 1 μM Olaparib and 1 μM PARGi PDD00017273 for 5 min and washed with PBS, then fixed with 4% paraformaldehyde (PFA, Sigma) for 15 min supplemented with 1 μM Olaparib and 1 μM PARGi PDD00017273, washed with PBS, permeabilized with 0.2% Triton X-100/PBS for 10 min and blocked with 10% FBS (GIBCO) for 30 min. Incubation with primary rabbit antibodies (anti-pan-ADPr, MABE1016, 1:500; anti-PARylation, ALX-210–890A-0100, 1:500; anti-MARylation AbD33204 (Bonfiglio et al., 2020), 1:500; anti-H3K9ac, 9649, 1:400) was performed for 2 h at room temperature followed by washing and 1-h incubation with Alexa Fluor 488-conjugated goat anti-rabbit secondary antibody (Molecular Probes/Thermo Fisher Scientific, A11034, 1:500). Coverslips were washed with PBS and counterstained with 0.1 μg/mL DAPI (4,6-diamidino-2-phenylindole, Sigma) in PBS for 10 min. After washing with PBS, coverslips were mounted onto glass slides with Mowiol 4–88 (Sigma). Images were acquired on Olympus Fluoview FV1200 confocal microscope using 40x/1.3, 60x/1.40 and 100x/1.40 Oil UPlanSApo objectives under non-saturating conditions. Image quantification was performed using CellProfiler (McQuin et al., 2018).

For the detection of APBs by immunofluorescence-FISH, after secondary antibody incubation, the immunofluorescence staining was fixed with 2% PFA for 10 min. PFA was washed off with PBS and coverslips dehydrated with successive washes in 70%, 95% and 100% ethanol for 3 min, and were allowed to air dry completely. Next, the coverslips were mounted on glass slides with hybridization mix (70% deionized formamide, 1 mg/mL of Blocking Reagent (Roche), 10 μM Tris-HCl pH 7.4) containing Alexa Fluor 488-(CCCTAA)_4_ PNA probe (PNA Bio). DNA was denatured by setting the slides on a heating block set to 72°C for 10 min and then incubating for at least 4 h or overnight at room temperature in the dark. The coverslips were then washed with Wash Solution A (70% deionized formamide and 10 μM Tris-HCl pH 7.2) and with Solution B (0.1 M Tris-HCl pH 7.2, 0.15 M NaCl and 0.08% Tween). Ethanol dehydration was repeated as above, and finally the samples were mounted and analyzed by conventional florescence with a × 40 and/or × 63 Plan λ objective (1.4 oil) using a Nikon 90i or Nikon A1R Spectral confocal microscope. Image were acquired using Nikon Ti2-E. Image quantification was performed using NIS-elements (Nikon).

#### Measurement of NAD^+^ levels

Cellular NAD^+^ levels were assayed using the NAD^+^/NADH Quantification Colorimetric Kit (BioVision) according to the manufacturer’s protocol. Briefly, U2OS cells were pre-treated with DMSO, 25 μM PARGi or 10 nM FK866 for 4 days. Cells were trypsinized, washed with ice-cold PBS and counted. 20,000 cells per sample were resuspended in NAD^+^/NADH Extraction Buffer and lysed by two repealed freeze thaw cycles on dry ice. Cells were vortexed and centrifuged at 14,000 rpm for 5 min to pellet cell debris. The supernatant was passed through a 10 kDa Spin Column (Abcam) at 10,000 g for 15 min to remove cellular enzymes that utilize NAD^+^/NADH as coenzymes. The supernatant from each condition was split into two to allow separate measurements of: 1) NADH-only and 2) NADt (NAD^+^ & NADH combined). To measure NADH-only, NAD^+^ was depleted by heating the samples to 60°C for 30 min. 100 μL of each sample or standard was placed in a white 96-well flat clear bottom plate (Corning). 100 μL NAD^+^ Cycling Enzyme Mix was added to each well before mixing on a plate shaker for 5 min. 10 μL of NADH Developer Solution was added to each well before mixing using a plate shaker for 15 min. The plate was incubated at room temperature for 30 min before measuring the absorbance 450 nm. NADH-only and NADt levels were calculated from absorbance values using a standard curve. NAD^+^ measurements were calculated by subtracting NADH-only values from NADt values. NAD_+_ measurements were normalized to the total protein content of each sample. Total protein content was determined using Bradford Protein Assay (Bio-Rad) according to the manufacturer’s protocol.

#### Transfection and immunoprecipitation

To obtain Figures S4C and S4D, control and ARH3 KO 293T cells were plated in 10-cm dishes with DMSO, 5 μM PDD00017273 or 5 μM PDD00017273 and 0.5 μM Olaparib. After 4 days, the cells were transfected with a plasmid expressing H3.1-GFP in the presence of inhibitors for 24 h using Polyfect (QIAGEN) following the manufacturer’s instructions. The cells were washed with PBS and lysed as described above. Protein concentrations were normalized, and the cell lysates were incubated with GFP-Trap MA magnetic agarose beads (ChromoTek) for 2 h while rotating at 4 °C. The beads were washed five times with Triton X-100 lysis buffer and eluted with 2x NuPAGE LDS sample buffer (Invitrogen) with TCEP (Sigma). The samples were then analyzed by western blotting. For Figures S4E and S4F, control and ARH3 KO 293T cells were plated and transfected 24 h later with a plasmid expressing YFP empty vector or PARP1-YFP and treated with 5 μM PARGi for 3 days. Then, the cells were trypsinized and counted using MOXI Z Automated Cell Counter, lysed with Triton X-100 lysis buffer and analyzed by western blotting.

#### Analysis of cell cycle and EdU incorporation

Cells were seeded in 6-well plates, treated and incubated with 10 μM EdU for 1 or 24 h at the end of treatment. Cells were harvested by trypsinization and labeled using the Click-iT Plus EdU Alexa Fluor 647 Flow Cytometry Assay Kit (Invitrogen) according to the manufacturer’s instructions. For DAPI staining, cell pellets were resuspended in 1 μg/mL DAPI solution in PBS and incubated protected from light for 10 min. Cells were washed in PBS and analyzed immediately after staining on Cytoflex LX (Beckman Coulter) or Cytek DxP8 (Becton Dickinson). Post-acquisition analysis was performed in FlowJo software (BD Biosciences).

#### PAR analysis by UPLC-MS/MS

UPLC-MS/MS analyses of PAR were performed as described previously (Martello et al., 2013; Zubel et al., 2017), with some modifications. Control and ARH3 KO U2OS cells were treated with DMSO or 25 μM PARGi for 4 days. Afterward, the media was removed, cells were washed with PBS and lysed by addition of ice-cold 20% TCA and detached mechanically using cell scrapers. Precipitates were pelleted by centrifugation at 3,000 g for 5 min, pellets washed with 70% ice-cold EtOH, air-dried for about 1 h at 37°C, and stored at −20°C until further processing. To detach protein-bound PAR, samples were dissolved in 255 μL 0.5 M KOH and subsequently neutralized by addition of 50 μL 4.8 M MOPS buffer. A 30 μL aliquot of the solution was stored at −80°C for DNA concentration determination. To the rest of the sample, 10 μL of 1.2 μM C^13^, N^15^-labeled PAR standard was added, and DNA and RNA were digested by adding 6.25 μL 2 M MgCl_2_, 2.5 μL 100 mM CaCl_2_, 12.5 μL 2 mg/mL DNase (Roche), and 2.5 μL 1 mg/mL RNase (Thermo Fisher Scientific), and incubated at 37°C for 3 h. Afterward, 1.25 μL of 40 mg/mL proteinase K (Roche) was added, and samples were incubated at 37°C overnight. PAR was purified using the High Pure microRNA Isolation kit (Sigma) according to the manufacturer’s instructions. To digest purified PAR to nucleosides, samples were incubated for 3 h at 37°C in a solution containing 10 U alkaline phosphatase (Sigma), 0.5 U phosphodiesterase (Fisher Scientific), 1.4 mM MgAc and 34 mM NH_4_Ac. Subsequently, ribosyl-adenosine (R-Ado) was quantified by isotope dilution UPLC-MS/MS using ACQUITY UPLC BEH C18 column, 130 Å, 1.7 μm and 2.1 mm × 50 mm (Waters) and ACQUITY UPLC H-Class coupled to a Xevo TQ-S triple quadrupole mass spectrometer (Waters). In parallel, DNA contents of samples were determined and used for normalization of R-Ado content to account for cell number variations. DNA samples were diluted in 180 μL dilution buffer (0.5 M KOH, 1 M MOPS). Standard curves were generated using calf thymus DNA (Sigma) denatured by addition of 1 M KOH, at 60°C for 30 min and subsequently neutralized by addition of 2 M MOPS buffer. Samples and standard solutions were then incubated with 5 μg/mL Hoechst 33342 (Invitrogen) for 5 min before being transferred to a 96-well plate. Fluorescence was measured with an excitation wavelength of 360/40 nm and an emission of 460/40 nm using a fluorescent plate reader.

#### *In vitro* ADP-ribosylation assays

To obtain Figure 2I, 2 μg of histone H3 (1–21) peptide or H3 (1–21) S10MAR peptide, a gift from Ivan Matic (Max Planck Institute for Biology of Aging), was incubated with 1 μM recombinant human PARP1 protein in reaction buffer (50 mM Tris-HCl, pH 8.0, 100 mM NaCl, 2 mM MgCl_2_, activated DNA and 50 μM NAD^+^ spiked with ^32^P-NAD^+^ from Perkin Elmer) at 37 °C for 30 min. For Figure S2H, 5 uM H3/H4 tetramer was incubated at room temperature for 20 min with recombinant human 100 nM PARP1 and 2 uM HPF1 proteins, 200 μM NAD^+^ and 500 nM DNA duplex (5′-ATCAGATAGCATCTGTGCGGCCGCTTAGGG-3′ and 5′-CCCTAAGCGGCCGCACA GATGCTATCTGAT-3′, Sigma) in the reaction buffer containing Mg^2+^ required by ARH3 (50 mM Tris, pH 7.5, 50 mM NaCl, and 2 mM MgCl_2_). Cold NAD^+^ was spiked with a small amount of ^32^P-NAD^+^. Under the reaction conditions, only H3 appears to be modified. The ADP-ribosylation reactions were stopped by addition of 2 μM Olaparib. ADP-ribose hydrolysis was started by adding recombinant human 1 μM ARH3 and/or PARG proteins and allowed to proceed at room temperature for 50 min. All concentrations are final, after mixing. Reactions were stopped by addition of 4x NuPAGE LDS sample buffer (Invitrogen) and incubation at 95 °C for 5 min. Samples were then analyzed by SDS-PAGE, including Instant Blue staining (Sigma), washing with water and gel drying, followed by autoradiography.

#### RNA-sequencing data generation and analysis

Control and ARH3 KO U2OS cells were treated with DMSO or 25 μM PARGi for 4 days. RNA was extracted using Direct-zol RNA MiniPrep Plus kit (Zymo Research) according to the manufacturer’s instructions. PolyA library preparation and RNA sequencing was performed by the Oxford Genomics Centre at the Wellcome Centre for Human Genetics. Briefly, RNA was quantified using Ribo-Green (Invitrogen) on the FLUOstar OPTIMA plate reader (BMG Labtech) and the size profile and integrity analyzed on the 2200 or 4200 TapeStation (Agilent, RNA ScreenTape). Input material was normalized to 100ng prior to library preparation. Polyadenylated transcript enrichment and strand specific library preparation was completed using NEBNext Ultra II Directional RNA library prep kit following manufacturer’s instructions. Libraries were amplified on a Tetrad (Bio-Rad) using in-house unique dual indexing primers based on Lamble et al. (2013). Individual libraries were normalized using Qubit, and the size profile was analyzed on the 2200 or 4200 TapeStation. Individual libraries were normalized and pooled together accordingly. The pooled library was diluted to ~10 nM for storage. The 10 nM library was denatured and further diluted prior to loading on the sequencer. Paired end sequencing was performed using a NovaSeq 6000 S4 Reagent Kit v1.5 (300 cycles). Adapters were trimmed with Cutadapt version 1.18 (Martin, 2011) in paired-end mode with the following parameters: -q 15, 10–minimum-length 10 -A AGATCGGAAGAGCGTCGTGTAGGGAAAGAGTGT -a AGATCGGAAGAGCACACGTCTGAACTCCAGTCA. Trimmed reads were first mapped to the ribosomal repeats to remove all the reads mapping to rDNA genes with STAR version 2.7.3a (Dobin et al., 2013) and the parameters:–runThreadN 16–readFilesCommand gunzip -c –k–outReadsUnmapped Fastx–limitBAMsortRAM 20000000000–outSAMtype BAM SortedByCoordinate. The unmapped reads were mapped to the human hg38 reference genome with STAR and the parameters–runThreadN 16–readFilesCommand gunzip -c –k–limitBAMsortRAM 20000000000–outSAMtype BAM SortedByCoordinate. SAMtools version 1.9 (Li et al., 2009) was used to retain only properly mapped reads and produced strand-specific bam files. FPKM-normalized bigwig files were created with deepTools version 3.4.2 (Ramírez et al., 2016) bamCoverage tool with the parameters -bs 10 -p max–normalizeUsing RPKM. For differential expression analysis, the aligned reads were aggregated on the Gencode V31 annotation with HTseq-count version 0.11.3 (Anders et al., 2015) and the list of differentially expressed genes obtained with DESeq2 version 3.12 (Love et al., 2014), keeping only the genes with a fold change < —1.5 or > 1.5 and an adjusted p value of 0.05. The values for the correlation heatmap were obtained with deepTools multiBigwigSummary with the parameters bins and –bs 10000 followed by plotCorrelation–outFileCorMatrix and the parameters–corMethod pearson–skipZeros –removeOutliers. The list of normalized read count for each gene across each sample was obtained with DESeq2. Reactome pathways enrichment from the MSigDB database (c2.cp.reactome.v7.2.symbols.gmt) were performed with GSEA version 4.1.0 (Mootha et al., 2003; Subramanian et al., 2005) with the following parameters: number of permutations: 1000; permutation type: gene_set; enrichment statistic: weighted; metric for ranking genes: Signal2Noise.

### QUANTIFICATION AND STATISTICAL ANALYSIS

Prism 7 (GraphPad) was used for statistical analysis, where *p < 0.05, **p < 0.01, ***p < 0.001. Details of statistical analyses are described in the figure legends.

## Supplementary Material

MMC1

MMC2

## Figures and Tables

**Table:** KEY RESOURCES TABLE

REAGENT or RESOURCE	SOURCE	IDENTIFIER
Antibodies		

anti-PARylation (rabbit polyclonal)	Trevigen	Cat# 4336-BPC-100; RRID:AB_2721257
anti-pan-ADPr binding reagent (rabbit monoclonal)	Millipore	Cat# MABE1016; RRID:AB_2665466
anti-PARylation (rabbit polyclonal)	Enzo Life Sciences	Cat# ALX-210-890A-0100; RRID: N/A
anti-MARylation (rabbit monoclonal)	Bonfiglio et al., 2020	AbD33204
anti-H3S10/28MAR (human polyclonal)	Bonfiglio et al., 2020	AbD33644
anti-histone H3 (rabbit polyclonal)	Millipore	Cat#: 07-690; RRID:AB_417398
anti-PARG (mouse monoclonal)	Millipore	Cat# MABS61; RRID:AB_10806473
anti-ARH3/ADPRH (rabbit polyclonal)	Atlas Antibodies	Cat#: HPA027104; RRID:AB_1060133
anti-PARP1 (rabbit monoclonal)	Abcam	Cat#: ab32138; RRID:AB_777101
anti-PARP2 (rabbit polyclonal)	Millipore	Cat# MABE18; RRID:AB_10807040
anti-PARP3 (rabbit polyclonal)	Proteintech	Cat# 11289-1-AP; RRID:AB_2283392
anti-γH2AX (rabbit polyclonal)	Abcam	Cat# ab2893; RRID:AB_303388
anti-H3S10P (rabbit polyclonal)	Abcam	Cat#: ab5176; RRID:AB_304763
anti-H3K9me3 (rabbit polyclonal)	Abcam	Cat#: ab8898; RRID:AB_306848
anti-H3K27ac (rabbit polyclonal)	Abcam	Cat# ab4729; RRID:AB_2118291
anti-β-tubulin (rabbit polyclonal)	Abcam	Cat# ab6046; RRID:AB_2210370
anti-H3K27me3 (mouse monoclonal)	Abcam	Cat# ab6002; RRID:AB_305237
anti-H3K9ac (rabbit monoclonal)	Cell Signaling	Cat# 9649; RRID:AB_823528
anti-H2AX (rabbit monoclonal)	Cell Signaling	Cat# 7631; RRID:AB_10860771
anti-HPF1 (rabbit polyclonal)	Gibbs-Seymour et al., 2016	N/A
anti-GAPDH (mouse monoclonal)	Millipore	Cat# MAB374; RRID:AB_2107445
anti-laminA (rabbit polyclonal)	Abcam	Cat# ab26300; RRID:AB_775965
anti-GFP (rabbit polyclonal)	Abcam	Cat# ab290; RRID:AB_303395
anti-BRCA1 (mouse monoclonal)	Millipore	Cat# OP92; RRID:AB_2750876
anti-BRCA2 (mouse monoclonal)	Millipore	Cat# OP95; RRID:AB_2067762
anti-FEN1 (rabbit polyclonal)	Abcam	Cat# ab17994; RRID:AB_444168
anti-CXXC5 (rabbit polyclonal)	Cell Signaling	Cat# 84546; RRID:AB_2800040
anti-PML (mouse monoclonal)	Santa Cruz	Cat# sc-996; RRID:AB_628162
anti-Hsp70 (mouse monoclonal)	Abcam	Cat# ab2787; RRID:AB_303300
anti-cyclin E1 (mouse monoclonal)	Cell Signaling	Cat# 4129; RRID:AB_2071200
anti-cyclin A (rabbit monoclonal)	Abcam	Cat# ab32798; RRID:AB_731777
anti-cyclin B1 (mouse monoclonal)	Millipore	Cat# 05-373; RRID:AB_309701
anti-PRC1-phospho-T481 (rabbit monoclonal)	Abcam	Cat# ab62366; RRID:AB_944969
anti-PRC1 (rabbit polyclonal)	Gruneberg et al., 2006	N/A
Goat polyclonal anti-mouse, HRP-conjugated	Agilent	Cat# P0447; RRID:AB_2617137
Swine polyclonal anti-rabbit, HRP-conjugated	Agilent	Cat# P0399; RRID:AB_2617141
Goat polyclonal anti-human, HRP-conjugated	Bio-Rad	Cat# STAR126P; *RRID*:AB_1605087
Goat polyclonal anti-rabbit, Alexa Fluor 488-conjugated	Thermo Fisher Scientific	Cat# A-11034; RRID:AB_2576217

Biological samples		

Control primary human fibroblasts	This paper	N/A
ARH3 C26F patient-derived primary human fibroblasts	This paper	N/A

Chemicals, peptides, and recombinant proteins		

PARG inhibitor PDD00017273	Sigma	Cat# SML1781
Olaparib	Cayman Chemical	Cat# 10621
Veliparib	Enzo Life Sciences	Cat# ALX-270-444-M005
Thymidine	CalBiochem	Cat# 6060
PolyFect Transfection Reagent	QIAGEN	Cat# 301105
TransIT-LT1 Transfection Reagent	Mirus Bio	Cat# MIR 2300
Puromycin	InvivoGen	Cat# ant-pr-1
Blasticidin	InvivoGen	Cat# ant-bl-1
cOmplete, EDTA-free Protease Inhibitor	Sigma	Cat# 11873580001
Cocktail		
PhosSTOP	Sigma	Cat# 4906845001
Benzonase	Sigma	Cat# 1016970001
4x NuPAGE LDS sample buffer	Invitrogen	Cat# NP0007
TCEP	Sigma	Cat# 646547
NuPAGE Novex 4-12% Bis-Tris gel	Invitrogen	Cat# WG1402A
Trichostatin A	Sigma	Cat# T8552
Formic acid LC/MS grade	Honeywell Fluka	Cat#15667520
Acetonitrile LC/MS grade	ROTISOLV	Cat# AE70.2
G-148 solution	Sigma	Cat# G8168
Activated DNA	Trevigen	Cat# 4671-096-06
NAD^+^	Trevigen	Cat# 4684-096-02
^32^PNAD^+^	Perkin Elmer	Cat# BLU023X250UC
Histone H3 peptide (1-21) Ac-ARTKQTARKS TGGKAPRKQLAGGK(Biotin)-Am	AnaSpec	Cat# AS-61702
Histone H3 (1–21) S10MAR peptide Ac-	A gift from Ivan Matic	N/A
ARTKQTARKS(ADPr)		
TGGKAPRKQLAGGK(Biotin)-Am		
		
Recombinant human PARP1 protein	Langelier et al., 2011	N/A
Recombinant human H3/H4 tetramer	Mehrotra et al., 2011	N/A
Recombinant human HPF1 protein	Gibbs-Seymour et al., 2016	N/A
Recombinant human ARH3 protein	Fontana et al., 2017	N/A
Recombinant human PARG protein	Dunstan et al., 2012	N/A
Alkaline phosphatase	Sigma	Cat# 10713023001
Phosphodiesterase	Fisher Scientific	Cat# 15838401
Calf thymus DNA	Sigma	Cat# D4764
DAPI	Sigma	Cat# D9542
Hoechst 33342	Invitrogen	Cat# H3570

Deposited data		

RNA-sequencing data	This study	GEO: GSE167060
Original imaging data	This study	https://doi.org/10.17632/zbmchm3fz4.1

Critical commercial assays		

NAD^+^/NADH Quantification Colorimetric Kit	BioVision	Cat# K337
QuikChange Lightning Site-Directed	Agilent	Cat# 210519
Mutagenesis Kit		
Lipofectamine RNAiMAX Reagent	Invitrogen	Cat# 13778150
PolyFect Transfection Reagent	QIAGEN	Cat# 301105
*Trans*IT-LT1 Transfection Reagent	Mirus Bio	Cat# MIR 2305
GFP-Trap Magnetic Agarose	Chromotek	Cat# gtma-20
Click-iT Plus EdU Alexa Fluor 647 Flow	Invitrogen	Cat# C10419
Cytometry Assay Kit		
Tel C-Alexa Fluor 488 PNA probe	PNA Bio	Cat# F1004
High Pure microRNA Isolation kit	Sigma	Cat# 5080576001
Subcellular Protein Fractionation kit for Cultured Cells	Thermo Fisher Scientific	Cat#78840
Direct-zol RNA Miniprep Plus Kit	Zymo Research	Cat# R2071
NEBNext Ultra II Directional RNA library prep kit	New England Biolabs	Cat# E7765
NovaSeq 6000 S4 Reagent Kit v1.5	Illumina	Cat# 20028312
LR Clonase II enzyme mix	Invitrogen	Cat# 11791020

Experimental models: cell lines		

Human: U2OS cells	ATCC	Cat# HTB-96
Human: U2OS ARH3 KO cells	Fontana et al., 2017	N/A
Human: U2OS ARH3 KO cells	This paper	N/A
complemented with untagged ARH3 WT		
Human: U2OS ARH3 KO cells	This paper	N/A
complemented with untagged ARH3		
D77/78N		
Human: HeLa cells	ATCC	Cat# CCL-2
Human: HeLa ARH3 KO cells	This paper	N/A
Human: 293T cells	ATCC	Cat# CRL-3216
Human: 293T ARH3 KO cells	Hanzlikova et al., 2020	N/A
Human: SUM159PT cells	BioIVT	RRID:CVCL_5423
Human: SUM159PT ARH3 KO cells	This paper	N/A
Human: SUM149PT cells	BioIVT	RRID:CVCL_3422
Human: SUM149PT ARH3 KO cells	This paper	N/A
Human: U251 cells	Sigma	Cat# 09063001
Human: U251 ARH3 KO cells	This paper	N/A
ARH3 C26F patient-derived primary human fibroblasts complemented with untagged	This paper	N/A
ARH3 WT		
ARH3 C26F patient-derived primary human fibroblasts complemented with untagged	This paper	N/A
ARH3 D77/78N		

Oligonucleotides		

sgRNA 210	Invitrogen	N/A
GCGCTGCTCGGGGACTGCGT		
sgRNA 212 GGGCGAGACGTCTATAAGGC	Invitrogen	N/A
Silencer Select Negative Control No. 1 siRNA	Invitrogen	Cat# 4390843
Silencer Select HPF1 siRNA	Invitrogen	Cat# s29883
Silencer Select PARG siRNA	Invitrogen	Cat# s16159
Silencer Select Negative Control No. 2 siRNA	Invitrogen	Cat# 4390847
Silencer Select BRCA1 siRNA	Invitrogen	Cat# s458
BRCA2 siRNA	Dharmacon	Cat# D-003462-04
GAAGAAUGCAGGUUUAAUA		

Recombinant DNA		

pDONR221 (Gateway vector)	Invitrogen	Cat# 12536017
pDEST12.2 (Gateway vector)	Invitrogen	Cat# 11808-011
pDEST12.2-ARH3 WT (plasmid)	This paper	N/A
pDEST12.2-ARH3 D77/78N (plasmid)	This paper	N/A
pLX304 (plasmid)	Addgene	Cat# 25890
pLX304-ARH3 WT (plasmid)	This paper	N/A
pLX304-ARH3 D77/78N (plasmid)	This paper	N/A
pCMV-VSV-G (plasmid)	Addgene	Cat# 8485
pCMV-dR8.2 (plasmid)	Addgene	Cat #8455
H3.1-GFP (plasmid)	Hanzlikova et al., 2020	N/A
pDEST-YFP (Gateway vector)	Invitrogen	Cat# V35820
pDEST-YFP-PARP1 (plasmid)	Gibbs-Seymour et al., 2016	N/A

Software and algorithms		

ImageJ	NIH	N/A
FlowJo	BD Biosciences	N/A
CellProfiler	McQuin et al., 2018	N/A
Cutadapt v1.18	Martin, 2011	N/A
STAR v2.7.3a	Dobin et al., 2013	N/A
SAMtools v1.19	Li et al., 2009	N/A
deepTools v.3.4.2	Ramírez et al., 2016	N/A
HTseq-count v0.11.3	Anders et al., 2015	N/A
DESeq2 v3.12	Love et al., 2014	N/A
GSEA v.4.1.0	Mootha et al., 2003; Subramanian et al., 2005	N/A
Prism 7	GraphPad	N/A

## Highlights

- Chromatin serine-linked MARylation is constantly produced throughout the cell cycle
- ADP-ribosylation reactions consist of distinct initiation and elongation steps
- PARG and ARH3 suppression is synthetically lethal because of accumulation of PARylation
- ARH3 deficiency increases PARPi resistance that can be exploited therapeutically
