# Supplementary material for: Unrestrained poly-ADP-ribosylation provides insights into chromatin regulation and human disease
Source: Mol Cell. Author manuscript; Available in PMC 2021 Jul 27. (PMC8221567; doi:10.1016/j.molcel.2021.04.028)
Supplement: MMC1 [file NIHMS1709114-supplement-MMC1.pdf]

Molecular Cell, Volume *81*

Supplemental information

Unrestrained poly-ADP-ribosylation provides insights into chromatin regulation and human disease

Evgeniia Prokhorova, Thomas Agnew, Anne R. Wondisford, Michael Tellier, Nicole Kaminski, Danique Beijer, James Holder, Josephine Groslambert, Marcin J. Suskiewicz, Kang Zhu, Julia M. Reber, Sarah C. Krassnig, Luca Palazzo, Shona Murphy, Michael L. Nielsen, Aswin Mangerich, Dragana Ahel, Jonathan Baets, Roderick J. O'Sullivan, and Ivan Ahel

Figure S1. ARH3 deficiency leads to the enrichment of HPF1-dependent MARylation on chromatin throughout all stages of the cell cycle, Related to Figure 1

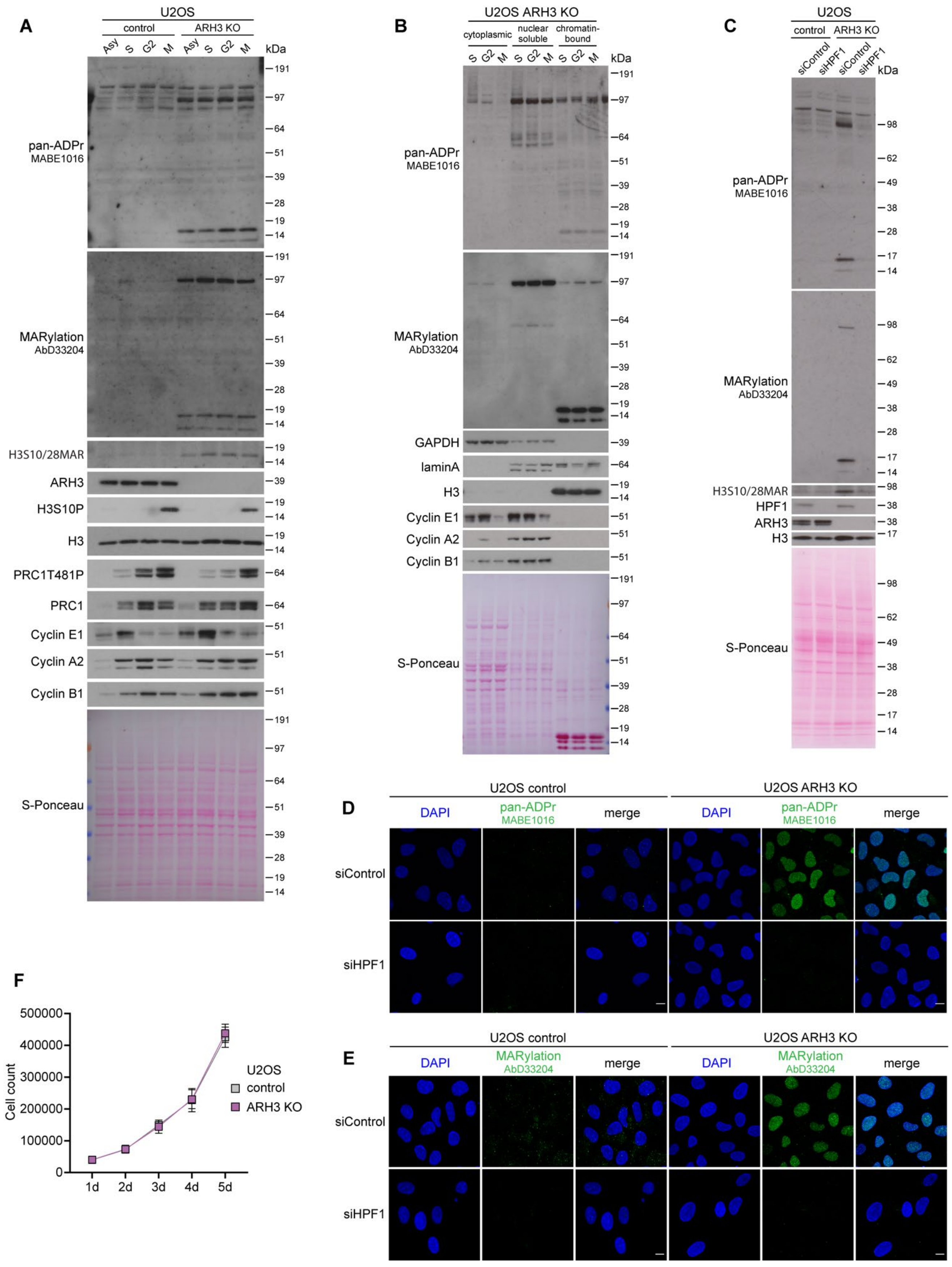

Figure S2. ARH3 and PARG have a synergistic effect on the accumulation of PARylation, Related to Figure 2

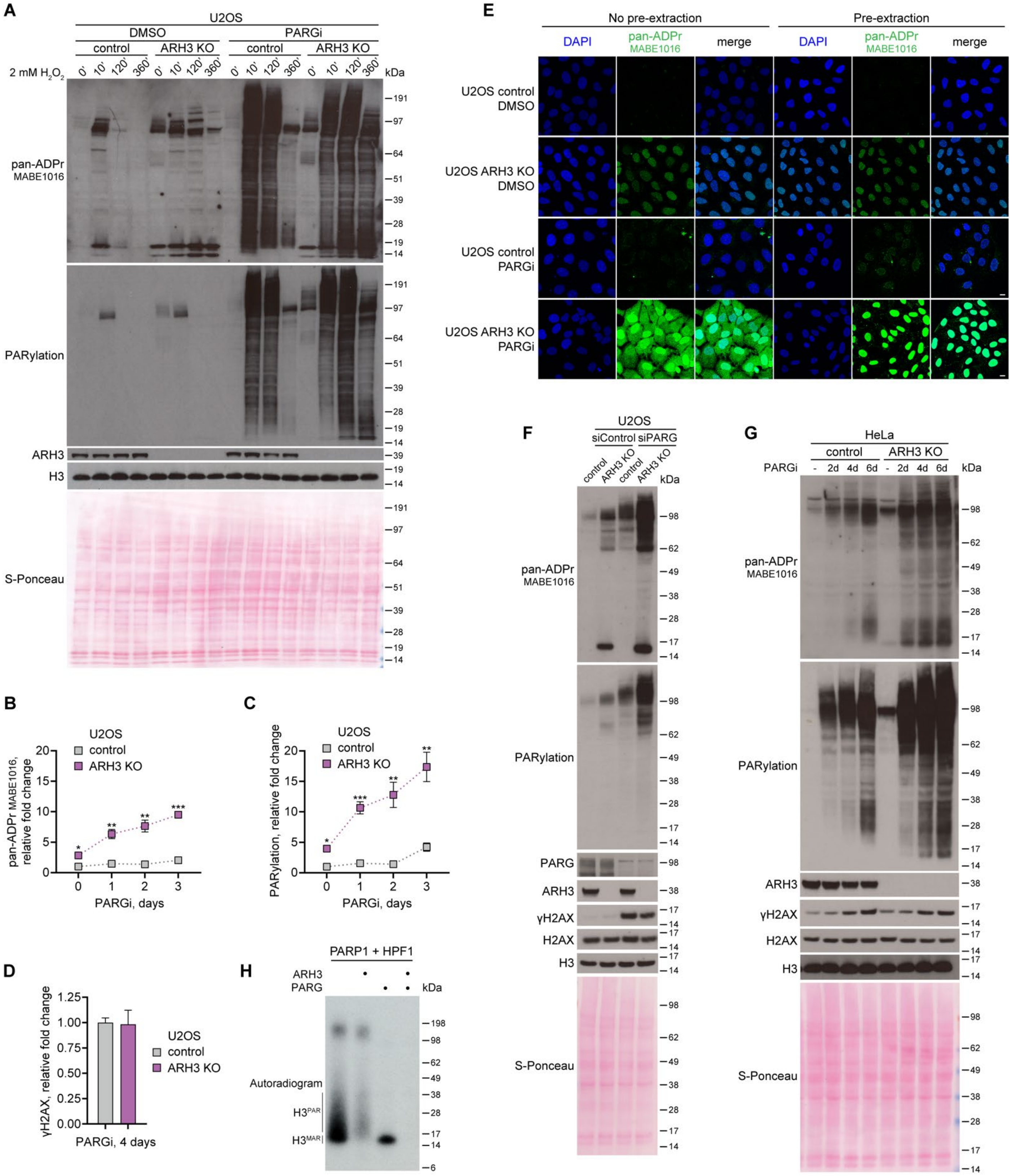

Figure S3. ARH3 activity modulates cancer cell sensitivity to PARG and PARP inhibition, Related to Figure 3

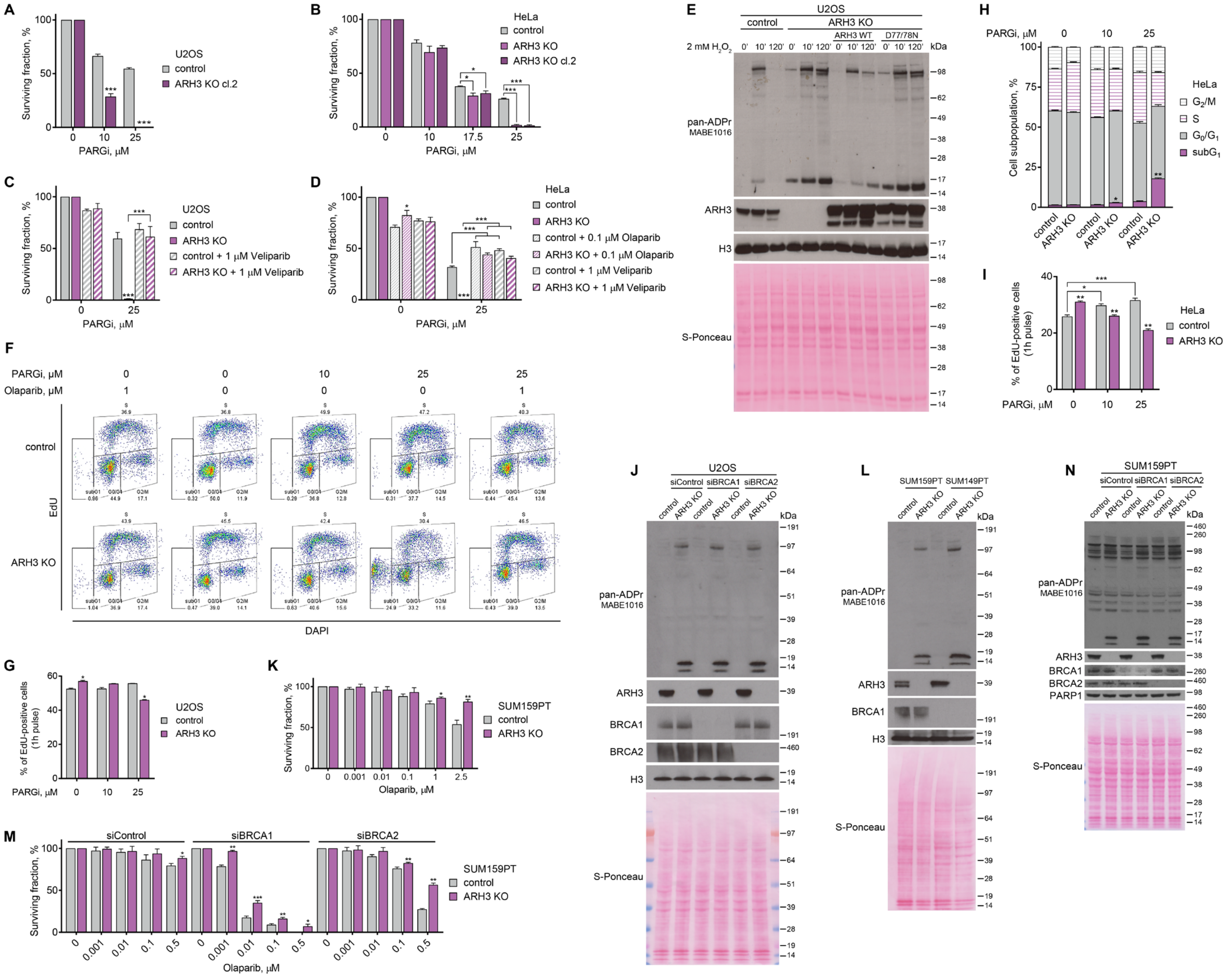

Figure S4. PARG inhibition combined with ARH3 deficiency leads to alterations in chromatin modification and transcription profiles, Related to Figure 4

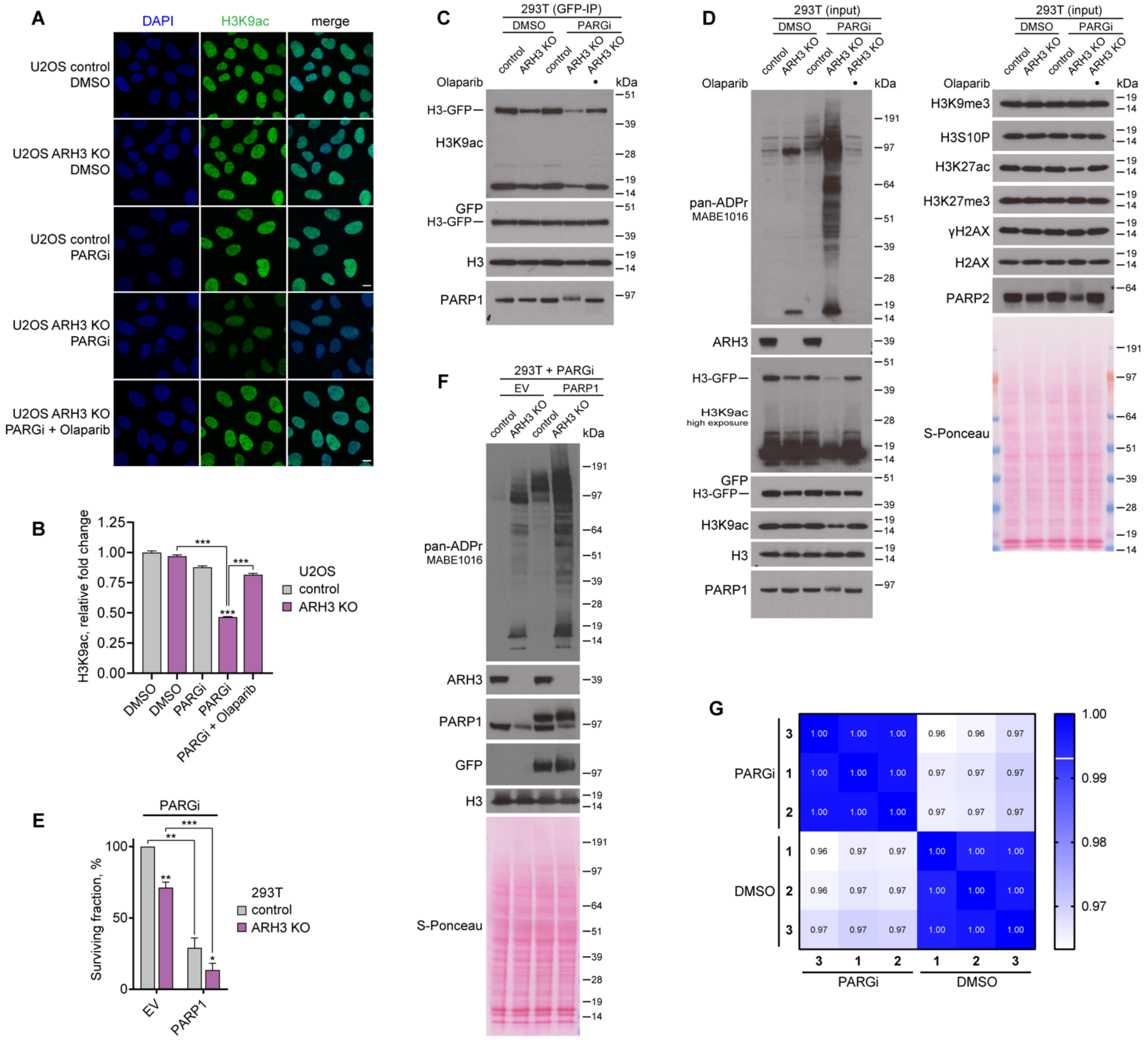

		PARGi 3	PARGi 1	PARGi 2	DMSO 1	DMSO 2	DMSO 3
PARGi	3	1.00	1.00	1.00	0.96	0.96	0.97
PARGi	1	1.00	1.00	1.00	0.97	0.97	0.97
PARGi	2	1.00	1.00	1.00	0.97	0.97	0.97
DMSO	1	0.96	0.97	0.97	1.00	1.00	1.00
DMSO	2	0.96	0.97	0.97	1.00	1.00	1.00
DMSO	3	0.97	0.97	0.97	1.00	1.00	1.00

Figure S5. PARG inhibition combined with ARH3 deficiency leads to excessive ADPr in patient-derived primary fibroblasts and glioblastoma cells, Related to Figure 5

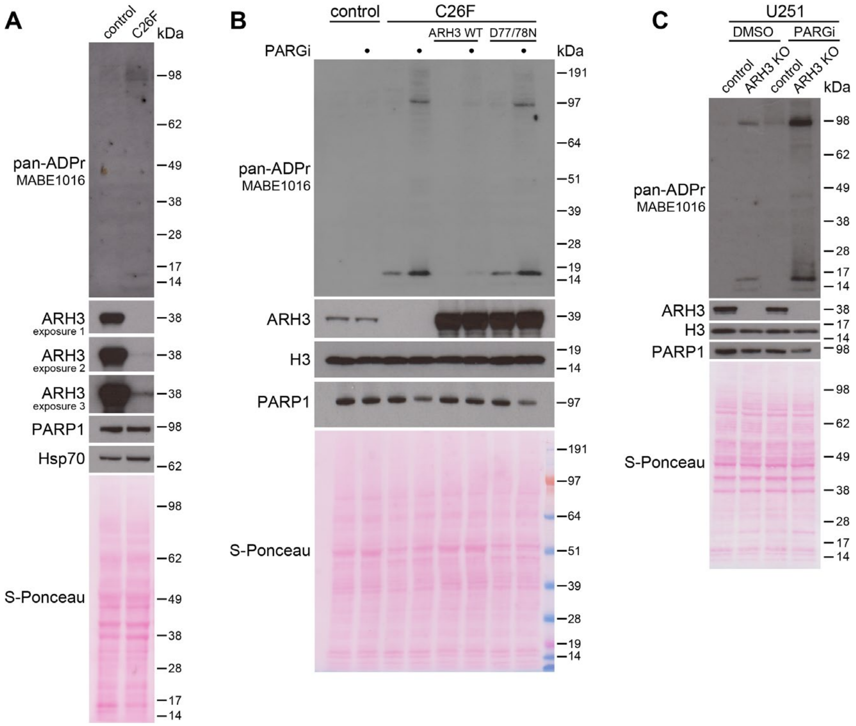

Supplemental Figure Legends

Figure S1. ARH3 deficiency leads to the enrichment of HPF1-dependent MARylation on chromatin throughout all stages of the cell cycle, Related to Figure 1

(A) Western blotting analysis of ADPr levels in control and ARH3 KO U2OS cells asynchronized (Asy) or synchronized by a double thymidine block and released for 4, 8 or 11 h (S, G2, and M, respectively).

(B) ARH3 KO U2OS cells were synchronized as in (A) and subjected to subcellular fractionation. ADPr signals were analysed by western blotting.

(C) Western blotting analysis of ADPr levels in control and ARH3 KO U2OS cells 72 h after PARG siRNA transfection.

(D and E) Pan-ADPr (D) and MARylation (E) signals were detected by immunofluorescence in detergent pre-extracted control and ARH3 KO U2OS cells 72 h after HPF1 siRNA transfection. Scale bars, 10 μm.

(F) Quantification of control and ARH3 KO U2OS cell proliferation by cell count every 24 h. Data are shown as mean±SD, n=3.

Figure S2. ARH3 and PARG have a synergistic effect on the accumulation of PARylation, Related to Figure 2

(A) Control and ARH3 KO U2OS cells were pre-treated with DMSO or 10 μM PARGi for 1 h followed by 2 mM H_2O_2 treatment in the presence of DMSO or 10 μM PARGi. ADPr signals were analyzed by western blotting at the indicated time points after H_2O_2 treatment. This panel is related to the Figure 7B in (Bartlett et al., 2018).

(B and C) Densitometry analysis of pan-ADPr (B) or PARylation (C) to histone H3 ratios detected by western blotting as in Figure 2B. Data represent fold change relative to DMSO-treated control cells and are shown as mean±SEM, n=3, *p<0.05, **p<0.01, ***p<0.001 (two-tailed Student's t-test).

(D) Densitometry analysis of γH2AX to H2AX ratios after 4 days of treatment with 25 μM PARGi detected by western blotting as in Figure 2B.

(E) Pan-ADPr signals were detected by immunofluorescence in detergent non-pre-extracted (left) and pre-extracted (right) control and ARH3 KO U2OS cells treated with DMSO or 25 μM PARGi for 6 days. Scale bars, 10 μm.

(F) Western blotting analysis of ADPr and γH2AX levels in control and ARH3 KO U2OS cells 72 h after PARG siRNA transfection.

(G) Control and ARH3 KO HeLa cells were treated with DMSO for 6 days or with 25 μM PARGi for the indicated number of days. ADPr and γH2AX levels were analyzed by western blotting.

(H) Radioactive ADP-ribosylation assay of histone H3 and subsequent hydrolysis by ARH3 and PARG alone or combined.

Figure S3. ARH3 activity modulates cancer cell sensitivity to PARG and PARP inhibition, Related to Figure 3

(A and B) Quantification of colony formation assay with control and an independent ARH3 KO U2OS clone (A) or with control and two independent ARH3 KO HeLa clones (B) treated with DMSO or PARGi. Data are shown as mean±SD, n=3, *p<0.05, ***p<0.001 (two-tailed Student's t-test).

(C and D) Quantification of colony formation assay with control and ARH3 KO U2OS (C) or HeLa (D) cells treated with DMSO, PARGi alone or in combination with PARPi. Data are shown as mean±SD, n=3, *p<0.05, ***p<0.001 (two-tailed Student's t-test).

(E) Control and ARH3 KO U2OS cells complemented with ARH3 WT or catalytically inactive ARH3 D77/78N were treated with H_2O_2 for the indicated times. ADPr signals were analyzed by western blotting.

(F) Representative scatterplots of cell cycle analysis by flow cytometry of EdU- and DAPI-stained cells after 6 day-exposure of control and ARH3 KO U2OS cells to DMSO or indicated treatment followed by 1-h EdU pulse.

(G) Cell proliferation and DNA synthesis in control and ARH3 KO U2OS cells after exposure to DMSO or PARGi for 2 days and 1-h EdU pulse. Data are shown as mean±SEM, n=2, *p<0.05 (two-tailed Student's t-test).

(H) Quantification of cell cycle analysis by flow cytometry of EdU- and DAPI-stained control and ARH3 KO HeLa cells after exposure to DMSO or PARGi for 6 days and 1-h EdU pulse. Data are shown as mean±SEM, n=3, *p<0.05, **p<0.01 (two-tailed Student's t-test).

(I) Cell proliferation and DNA synthesis in control and ARH3 KO HeLa cells after exposure to DMSO or PARGi for 6 days and 1-h EdU pulse. Data are shown as mean±SEM, n=3, *p<0.05, **p<0.01, ***p<0.001 (two-tailed Student's t-test).

(J and N) Western blotting analysis of ADPr signals in control and ARH3 KO U2OS (J) or SUM159PT (N) cells 72 h after BRCA1 or BRCA2 siRNA transfection.

(K and M) Quantification of colony formation assay with control and ARH3 KO SUM159PT cells treated with DMSO or Olaparib alone (K) or after BRCA1 or BRCA2 siRNA transfection (M). Data are shown as mean±SD, n=3, *p<0.05, **p<0.01, ***p<0.001 (two-tailed Student's t-test).

(L) Western blotting analysis of ADPr signals in control and ARH3 KO SUM159PT and SUM149PT cells.

Figure S4. PARG inhibition combined with ARH3 deficiency leads to alterations in chromatin modification and transcription profiles, Related to Figure 4

(A) Control and ARH3 KO U2OS cells were treated with DMSO, 25 μM PARGi or 25 μM PARGi and 1 μM Olaparib for 6 days. H3K9ac levels were compared by immunofluorescence. Scale bars, 10 μm.

(B) Quantification of immunofluorescence as in Figure 4C. Data represent fold change relative to DMSO-treated control cells±SEM, at least 500 cells were analyzed per condition, ***p<0.001 (one-way ANOVA followed by Tukey post-test).

(C and D) Control and ARH3 KO 293T cells were treated with DMSO, 5 μM PARGi or 5 μM PARGi and 0.5 μM Olaparib for 4 days and transfected with H3-GFP in the presence of inhibitors for 24 h. H3-GFP immunoprecipitation (GFP-IP, C) and input samples (D) were analyzed be western blotting for ADPr, H3 modification, γH2AX, PARP1/2 levels.

(E and F) Control and ARH3 KO 293T cells were transfected with YFP empty vector (EV) or PARP1-YFP and treated with 5 μM PARGi for 3 days. Quantification of cell survival by cell count (E) and western blot analysis of ADPr and PARP1 levels (F) are shown. Data in (E) represent mean±SEM, n=4, *p<0.05, **p<0.01, ***p<0.001 (two-tailed Student's t-test).

(G) Heat map of Pearson's correlation coefficients used to measure reproducibility of sample preparation and sequencing results between three independent replicates of ARH3 KO U2OS cells treated with DMSO or 25 μM PARGi for 4 days.

Figure S5. PARG inhibition combined with ARH3 deficiency leads to excessive ADPr in patient-derived primary fibroblasts and glioblastoma cells, Related to Figure 5

(A) Western blotting analysis of ADPr and ARH3 levels in control and ARH3 C26F mutant patient-derived primary human fibroblasts.

(B) Control and ARH3 C26F mutant patient-derived primary human fibroblasts complemented with ARH3 WT or catalytically inactive ARH3 D77/78N were treated with 25 μM PARGi for 4 days. ADPr and PARP1 signals were analyzed by western blotting.

(C) Western blotting analysis of ADPr and PARP1 levels in control and ARH3 KO U251 cells after 4-day treatment with DMSO or 25 μM PARGi.
